# Gut microbiota and risk of five common cancers: A univariable and multivariable Mendelian randomization study

**DOI:** 10.1002/cam4.5772

**Published:** 2023-03-07

**Authors:** Zixin Wei, Biying Yang, Tiantian Tang, Zijing Xiao, Fengzhan Ye, Xiaoyu Li, Shangbin Wu, Jin‐gang Huang, Shanping Jiang

**Affiliations:** ^1^ Department of Pulmonary and Critical Care Medicine, Sun Yat‐Sen Memorial Hospital Sun Yat‐Sen University Guangzhou Guangdong China; ^2^ Department of Neurology, Sun Yat‐Sen Memorial Hospital Sun Yat‐Sen University Guangzhou Guangdong China; ^3^ Department of Neurology, Guangdong Provincial Hospital of Chinese Medicine The Second Affiliated Hospital of Guangzhou University of Chinese Medicine Guangzhou Guangdong China; ^4^ Guangzhou Medical University Guangzhou Guangdong China; ^5^ Department of Pediatrics, Nanfang Hospital Southern Medical University Guangzhou Guangdong China; ^6^ Department of Pediatrics, Guangdong Provincial Hospital of Chinese Medicine The Second Affiliated Hospital of Guangzhou University of Chinese Medicine Guangzhou Guangdong China; ^7^ Medical Research Center, Sun Yat‐Sen Memorial Hospital Sun Yat‐Sen University Guangzhou Guangdong China

**Keywords:** cancer, causality, gut microbiota, Mendelian randomization

## Abstract

**Background:**

Previous studies have linked gut microbiota with cancer etiology, but the associations for specific gut microbiota are causal or owing to bias remain to be elucidated.

**Methods:**

We performed a two‐sample Mendelian randomization (MR) analysis to assess the causal effect of gut microbiota on cancer risk. Five common cancers, including breast, endometrial, lung, ovarian, and prostate cancer as well as their subtypes (sample sizes ranging from 27,209 to 228,951) were included as the outcomes. Genetic information for gut microbiota was obtained from a genome‐wide association study (GWAS) comprising 18,340 participants. In univariable MR (UVMR) analysis, the inverse variance weighted (IVW) method was conducted as the primary method, with the robust adjusted profile scores, weighted median, and MR Egger used as supplementary methods for causal inference. Sensitivity analyses including the Cochran Q test, Egger intercept test, and leave‐one‐out analysis were performed to verify the robustness of the MR results. Multivariable MR (MVMR) was performed to evaluate the direct causal effects of gut microbiota on the risk of cancers.

**Results:**

UVMR detected a higher abundance of genus *Sellimonas* predicted a higher risk of estrogen receptor‐positive breast cancer (OR = 1.09, 95% CI 1.05–1.14, *p* = 2.01 × 10^−5^), and a higher abundance of class *Alphaproteobacteria* was associated with a lower risk of prostate cancer (OR = 0.84, 95% CI 0.75–0.93, *p* = 1.11 × 10^−3^). Sensitivity analysis found little evidence of bias in the current study. MVMR further confirmed that genus *Sellimonas* exerted a direct effect on breast cancer, while the effect of class *Alphaproteobacteria* on prostate cancer was driven by the common risk factors of prostate cancer.

**Conclusion:**

Our study implies the involvement of gut microbiota in cancer development, which provides a novel potential target for cancer screening and prevention, and might have an implication for future functional analysis.

## Graphical abstract

We found that sarcopenia and cachexia have different effects on OS for PDAC patients undergoing radical excision by studying the data of 614 PDAC patients.

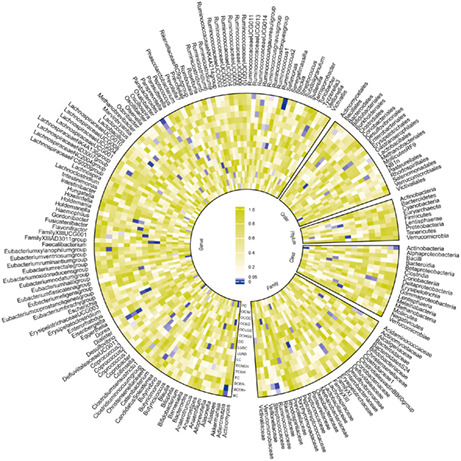

## 1 INTRODUCTION

The global burden of cancer incidence and mortality is rapidly growing.^1^ It has been predicted that the global cancer burden in the next 20 years would rise by nearly 50%.^2^ According to Cancer Statistics 2022, prostate cancer alone accounts for 27% of diagnoses in men and breast cancer accounts for almost one‐third in women, while lung cancer remains the leading cause in terms of cancer deaths in both male and female.^3^ Given the enormous threat brought to human health and the accompanying economic burden on human beings caused by cancer, early cancer screening and prevention is of great importance.^4^

Within the past 15 years, investigation of the gut microbiota in the issue of human health has increased exponentially.^5^ Disruption of gut microbiota balance has been linked with various disease states, like obesity, psychiatry disorders, and autoimmunity diseases.^6^, ^7^, ^8^ A majority of the literature has also reported the potential influence of gut microbiota exerting on human health via microbiota‐derived metabolites, modulation of host immunity and metabolism.^9^, ^10^, ^11^, ^12^ Hence, there might be tight contact of dysbiosis with host health conditions, for which dysbiosis might act as a cause or consequence.

To date, accumulation of evidence has implicated potential associations between gut microbiota profiles and cancer risk.^13^ If such associations are causal, then gut microbiota might be a novel target for cancer screening and prevention. Previous studies, based on animal models, have reported gut microbiota participated in tumor development through various signal pathways.^14^, ^15^ Observational studies also supported the involvement of gut microbiota in certain cancers. In a case–control study, Goedert et al. observed altered composition of gut microbiota in patients with breast cancer.^16^ Another prospective study found a higher abundance of *Bacteroides massiliensis* in patients with prostate cancer compared to healthy controls.^17^ Nevertheless, a conclusive cancer‐causing microbiota community composition has not been determined. Notably, it is not yet enough to draw a firm conclusion about the potential causality between gut microbiota and cancer risk based on the existing evidence derived from observational studies. Conventional observational studies were limited by inherent defects, including resident confounding, reverse causality, and inadequate attention to variation by histology of cancers.^18^ Besides, it should be noted that some treatments, like antibiotic usage, chemotherapy, and surgery, could also influence the profiles of host gut microbiota, leading to a tremendous impact on the accuracy of the results.^19^, ^20^, ^21^ Therefore, it is difficult to distinguish whether bacterial disruption acted as a cause or consequence of cancer. The causal effect of gut microbiota on cancer risk remains to be elucidated. While randomized controlled trial (RCT) is the gold standard in determining causality, the long incubation period from certain microbiota exposure to oncogenesis makes it impractical in clinical settings.^22^ In this context, a novel way to investigate the causal effect of gut microbiota on cancer risk is warranted.

Mendelian randomization (MR) is a recently developed method typically used for causal inference.^23^ In MR, single nucleotide polymorphisms (SNPs) were utilized as unconfounded instrumental variants (IVs) to proxy exposure phenotypes.^24^ An MR design mimics an RCT since genetic variants are randomly allocated during fertilization, hence making confounding less likely.^25^, ^26^ In addition, genotype formation is prior to disease onset and is generally unaffected by disease onset of progression, thereby less vulnerable to reverse causality. In this study, we performed a two‐sample multivariable MR study to investigate the causal effect of genetically predicted gut microbiota on five common cancers, including breast cancer (BC), endometrial cancer (EC), lung cancer (LC), ovarian cancer (OC), prostate cancer (PC), and their histologic subtypes.

## 2 METHODS

### 2.1 Study design

**FIGURE 1:**
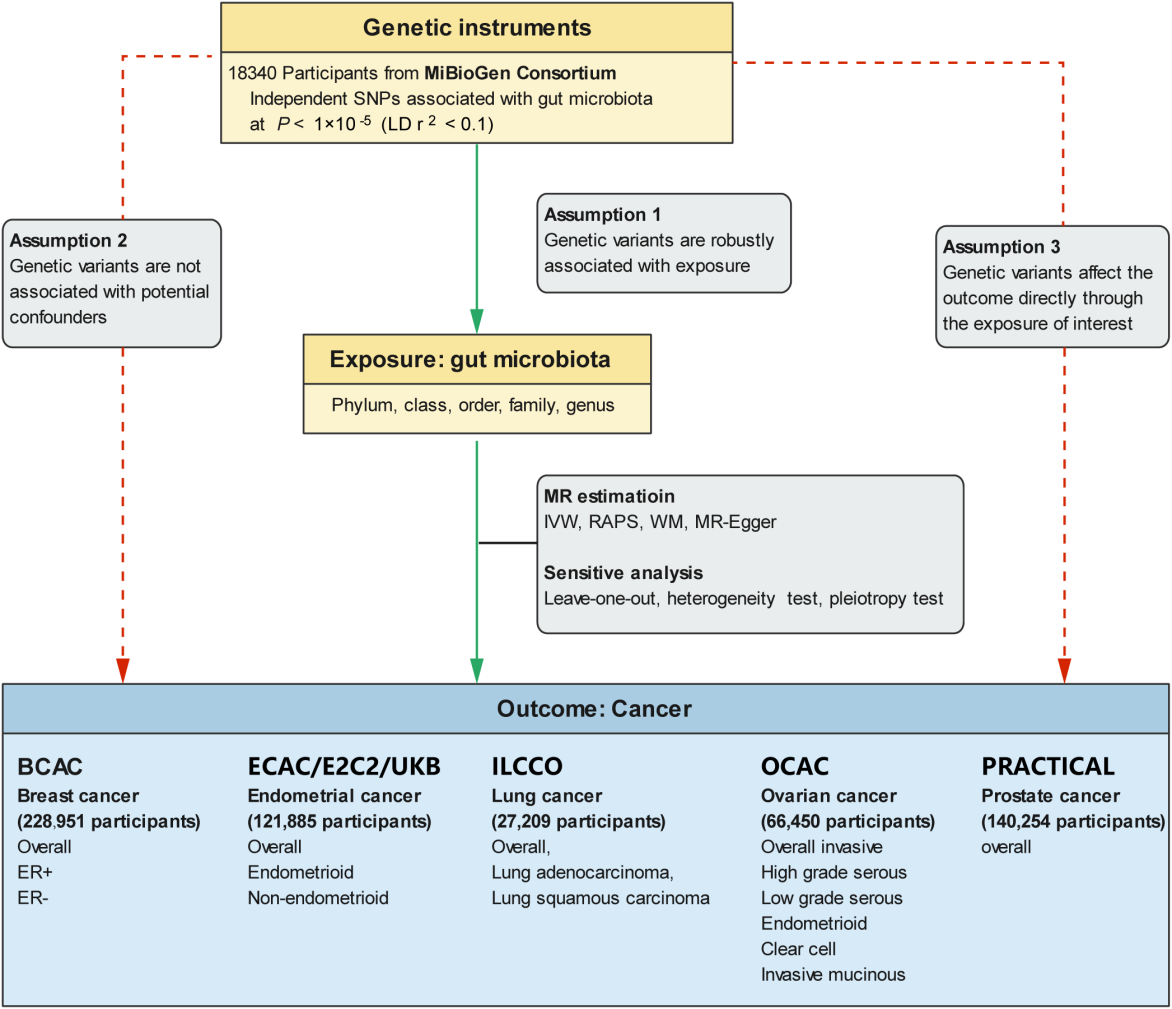
Overview of the current Mendelian randomization (MR) study. BCAC, Beast Cancer Association Consortium; E2C2, Epidemiology of Endometrial Cancer Consortium; ECAC, Endometrial Cancer Association Consortium; ER‐, estrogen receptor‐negative; ER+, estrogen receptor‐positive; ILLCO, International Lung Cancer Consortium; OCAC, Ovarian Cancer Association Consortium; PRACTICAL, Prostate Cancer Association Group to Investigate Cancer‐Associated Alterations in the Genome Consortium. SNP, single nucleotide polymorphism; UKB, United Kingdom Biobank.

Leveraging a two‐sample Mendelian randomization framework, we assessed the causal effect of gut microbiota on five common cancers, including BC, EC, LC, OC, and PC as well as their subtypes. To comprehensively investigate the role of gut microbiota in the issue of cancer incidence, the MR analyses were conducted at five distinct feature levels, including phylum, class, order, family, and genus. The study design accompanied by the fundamental MR assumptions was presented in Figure 1.

### 2.2 Data sources

The genetic information of gut microbiota was accessed from a largest GWAS conducted by the MiBioGen consortium, comprising 18,340 participants from 24 cohorts (~78% Europeans).^27^ Totally 211 taxa were included in the GWAS (9 phyla, 16 classes, 20 orders, 36 families, and 131 genera). Specifically, three different regions within the 16 S rRNA gene were targeted to profile the microbial composition. Before exploring the effect of host genes on the abundance of bacterial taxa, age, sex, technical covariates, and genetic principal components were adjusted. Further information about the microbiota data was described elsewhere,^27^ and the GWAS data could be obtained at https://mibiogen.gcc.rug.nl/.

The summary GWAS data for BC was obtained from the Breast Cancer Association Consortium (BCAC) consisting of 122,977 cases and 105,974 controls, and all the participants were of European ancestry.^28^ Given that summary statistics of the two subtypes [estrogen receptor‐positive (ER+) and negative (ER‐) BC] were also available, subgroup analysis was performed.

Summary data for EC and its histologic subtypes (endometrioid and non‐endometrioid subtypes) were obtained from a large‐scale GWAS consisting of the Endometrial Cancer Association Consortium (ECAC), the Epidemiology of Endometrial Cancer Consortium (E2C2), and the UK Biobank, involving up to 12,906 cases and 108,979 health controls from European ancestry.^29^

We obtained summary‐level statistics of LC from the International Lung Cancer Consortium (ILCCO) comprising 11,348 patients with lung cancer and 15,861 controls (all were European descents).^30^ The genetic information of the two histologic subtypes, including lung adenocarcinoma (LUAD) and lung squamous cell carcinoma (LUSC), was extracted for MR subgroup analysis.

For overall invasive epithelial ovarian cancer, we used the GWAS statistics from the Ovarian Cancer Association Consortium (OCAC), comprising up to 25,509 cases and 40,941 controls of European ancestry.^31^ We also conducted subgroup analysis for the histologic subtypes, including high‐grade serous, low‐grade serous, endometrioid, clear cell, and invasive mucinous ovarian cancer.

The associations of SNPs with PC were obtained from the GWAS study from the Prostate Cancer Association Group to Investigate Cancer‐Associated Alterations in the Genome (PRACTICAL) Consortium, consisting of 79,148 cases diagnosed with prostate cancer and 61,106 controls of European descent.^32^ Only overall PC GWAS statistics were publicly available without any application.

Specifically, the GWAS data for these cancers were deposited at the Integrative Epidemiology Unit (IEU) Open GWAS Project (https://gwas.mrcieu.ac.uk/).

The GWAS summary data for the traditional risk factors of BC and PC, including age at menarche, age at menopause, body mass index, alcoholic drinking, and regular smoking, were obtained from corresponding consortia.^33^, ^34^, ^35^, ^36^

### 2.3 Instruments selection

211 bacterial taxa were grouped into five taxonomic levels (phylum, class, order, family, and genus). Fifteen bacterial taxa were unknown and thereby were excluded from our study, making 196 bacterial taxa remained. Considering the limited number of SNPs available, we used the SNPs at a lenient *p*‐value <1 × 10^−5^, which was widely used in the case of the limited number of SNPs available.^37^, ^38^ Concordant with previous research, we clumped the genetic variants within 500 kb at the threshold of linkage disequilibrium (LD) *r* ^2^ < 0.1, based on European ancestry reference data from the 1000 Genomes Project.^39^ F‐statistic for each SNP was calculated to quantify the statistical strength, and those with an F statistic <10 were discarded. The calculation of F‐statistic was described in detail elsewhere.^37^ Then, SNPs were retrieved and extracted from the outcome data, and the SNPs significantly associated with the outcomes (*p* < 1 × 10^−5^) were eliminated. If SNPs could not be found in the outcome datasets, proxies at the threshold of LD *r* ^2^ > 0.8 were used if applicable. Finally, we aligned the effect alleles of the exposure‐ and outcome‐SNPs through harmonization, and excluded those with incompatible alleles (e.g., A/C paired with A/G) or being palindromic with intermediate allele frequency.

### 2.4 Statistical analysis

For Univariable MR analysis (UVMR), the random‐effects inverse‐variance weighted (IVW) method was performed as the primary analysis for causality inference, for which the Wald ratio estimates were combined to elicit a pooled effect on the outcome.^40^ Several alternative models, including robust adjusted profile score (RAPS), weighted median, and MR‐Egger, were utilized to evaluate the robustness of the MR results.^41^, ^42^, ^43^ Specifically, the RAPS method is relatively robust when existing weak instruments.^44^ The weighted median method hypothesizes that less than 50% of the SNPs are invalid, and the statistical power is mildly weaker than the IVW method.^45^ For MR‐Egger, the power is weak and typically used for direction evaluation.^46^

For the primary MR results, multiple‐testing significance was determined at each feature level using Bonferroni correction (*p* < 0.05/*n*, where *n* is the number of bacterial taxa included in each feature level). Hence, the multiple‐testing significance was 5.56 × 10^−3^, 3.13× 10^−3^, 2.5 × 10^−3^, 1.56 × 10^−3^, and 4.20 × 10^−4^, respectively, for phylum, class, order, family, and genus. We also considered a nominal significance level for the MR estimates at *p* < 0.05.

For both significant and nominal significant causalities, we conducted sensitivity analyses using a series of statistical methods to detect whether the MR assumptions were violated. The leave one out (LOO) analysis was undertaken to appraise whether the pooled estimation was biased by any high‐influence point.^47^ The Cochran Q test was conducted to evaluate heterogeneity.^48^ The intercept term derived from MR‐Egger regression was used to detect horizontal pleiotropy.^43^

Given that the composition of gut microbiota could be influenced by endocrine factors and diet habits, UVMR could not reflect a direct effect of gut microbiota on cancer incidence as these endocrine factors or diet habits could also influence cancer risk. To determine whether the observed significant effect of bacterial taxa on cancer was a direct or indirect impact, we further conducted multivariable MR analysis (MVMR) accounting for the traditional risk factors of the cancers.^49^ Similarly, IVW, WM, and MR‐Egger regression were used for analysis, and the intercept derived from MR‐Egger regression was used to detect potential horizontal pleiotropy.

All analyses were performed based on the R program (version 4.0.0) using the “TwoSampleMR” package (version 0.5.4) and the “Mendelian Randomization” package (version 0.5.1).

## 3 RESULTS

In total, 196 bacterial taxa (9 phyla, 16 classes, 20 orders, 32 families, and 119 genera) were included for MR analysis (Table S1). After rigorous instrument selection steps, the number of SNPs associated with each of the bacterial taxa ranged from three to 22 (Table 1). All F‐statistics were over 10, suggesting no weak instrumental variables were employed (Table S1).

**TABLE 1:** Significant and nominal significant MR results.

Cancer	Gut microbiota	SNP (*n*)	IVW		RAPS		WM		MR‐Egger	
			OR (95% CI)	*p*	OR (95% CI)	*p*	OR (95% CI)	*p*	OR (95% CI)	*p*
BC	Class. *Actinobacteria*	22	0.94 (0.89, 0.99)	0.02	0.95 (0.90, 0.996)	0.03	0.95 (0.89, 1.01)	0.09	0.99 (0.86, 1.13)	0.85
	Genus. *Parabacteroides*	5	0.87 (0.79, 0.96)	7.31 × 10^−3^	0.87 (0.78, 0.98)	0.02	0.89 (0.78, 1.01)	0.08	0.91 (0.69, 1.22)	0.59
	Genus. *Sellimonas*	11	1.05 (1.02, 1.09)	2.31 × 10^−3^	1.06 (1.02, 1.10)	4.86 × 10^−3^	1.03 (0.98, 1.08)	0.21	1.01 (0.86, 1.20)	0.09
BC (ER+)	Genus. *Adlercreutzia*	8	0.88 (0.81, 0.95)	7.16 × 10^−4^	0.88 (0.81, 0.96)	2.81 × 10^−3^	0.90 (0.81, 0.99)	0.03	1.01 (0.72, 1.42)	0.96
	Genus. *Sellimonas*	11	1.09 (1.05, 1.14)	2.01 × 10^−5^	1.09 (1.05, 1.14)	7.60 × 10^−5^	1.10 (1.04, 1.17)	1.24 × 10^−3^	1.03 (0.85, 1.25)	0.76
BC (ER‐)	Genus. *Dorea*	11	0.84 (0.73, 0.96)	8.95 × 10^−3^	0.84 (0.72, 0.97)	0.02	0.82 (0.69, 0.98)	0.03	0.80 (0.57, 1.12)	0.23
	Genus. *Eubacterium* *xylanophilum group*	9	0.82 (0.71, 0.95)	7.41 × 10^−3^	0.81 (0.69, 0.94)	4.42 × 10^−3^	0.80 (0.67, 0.97)	0.02	0.77 (0.47, 1.26)	0.34
	Genus. *Lachnospiraceae NK4A136 group*	15	0.88 (0.79, 0.97)	0.01	0.88 (0.79, 0.98)	0.02	0.86 (0.74, 0.98)	0.03	0.86 (0.70, 1.05)	0.17
EC (EH)	Genus. *Dorea*	11	0.74 (0.60, 0.91)	3.81 × 10^−3^	0.71 (0.56, 0.89)	3.28 × 10^−3^	0.73 (0.56, 0.95)	0.02	0.61 (0.35, 1.06)	0.11
	Genus. *Lactobacillus*	10	0.81 (0.71, 0.94)	4.01 × 10^−3^	0.81 (0.69, 0.95)	8.17 × 10^−3^	0.86 (0.72, 1.03)	0.10	0.74 (0.48, 1.15)	0.22
LC	Genus. *Erysipelatoclostridium*	16	1.19 (1.05, 1.36)	8.85 × 10^−3^	1.20 (1.04, 1.39)	0.01	1.19 (0.98, 1.44)	0.07	0.91 (0.53, 1.56)	0.73
	Genus. *Holdemanella*	11	1.22 (1.06, 1.40)	5.29 × 10^−3^	1.24 (1.03, 1.48)	0.02	1.23 (1.03, 1.47)	0.02	1.26 (0.83, 1.92)	0.30
LUAD	Family. *Peptococcaceae*	10	0.70 (0.55, 0.88)	2.51 × 10^−3^	0.70 (0.53, 0.92)	0.01	0.73 (0.53, 0.997)	0.05	1.05 (0.59, 1.85)	0.88
	Genus. *Erysipelatoclostridium*	16	1.37 (1.11, 1.68)	2.68 × 10^−3^	1.38 (1.10, 1.72)	5.46 × 10^−3^	1.42 (1.08, 1.87)	0.01	0.94 (0.41, 2.19)	0.89
	Genus. *Eubacterium* *Hallii group*	15	0.72 (0.56, 0.93)	0.01	0.71 (0.54, 0.94)	0.02	069 (0.49, 0.97)	0.03	0.83 (0.47, 1.49)	0.55
	Genus. *Gordonibacter*	12	0.82 (0.71, 0.94)	5.58 × 10^−3^	0.81 (0.69, 0.95)	8.82 × 10^−3^	0.80 (0.66, 0.97)	0.03	0.50 (0.27, 0.93)	0.05
LUSC	Phylum. *Tenericutes*	12	0.71 (0.56, 0.91)	6.00 × 10^−3^	0.70 (0.53, 0.92)	0.0.01	0.77 (0.56, 1.07)	0.12	1.08 (0.48, 2.39)	0.86
	Class. *Mollicutes*	12	0.71 (0.56, 0.91)	6.00 × 10^−3^	0.70 (0.53, 0.92)	0.0.01	0.77 (0.56, 1.07)	0.12	1.08 (0.48, 2.39)	0.86
	Genus. *Flavonifractor*	6	1.89 (1.23, 2.89)	3.73 × 10^−3^	2.00 (1.37, 2.93)	3.75 × 10^−4^	1.98 (1.25, 3.15)	3.69 × 10^−3^	1.17 (0.13, 10.47)	0.89
	Genus. *Ruminococcus1*	8	0.59 (0.40, 0.88)	0.01	0.61 (0.36, 1.02)	0.06	0.64 (0.38, 1.10)	0.11	1.38 (0.40, 4.74)	0.62
OC	Genus. *Family XIIIAD3011 group*	12	0.83 (0.72, 0.95)	6.50 × 10^−3^	0.82 (0.70, 0.95)	0.01	0.81 (0.68, 0.98)	0.03	0.65 (0.33, 1.29)	0.25
OC (HGS)	Family. *Alcaligenaceae*	15	0.80 (0.68, 0.95)	8.94 × 10^−3^	0.78 (0.66, 0.94)	8.38 × 10^−3^	0.76 (0.61, 0.94)	0.01	0.68 (0.33, 1.37)	0.30
	Genus. *Christensenellaceae* *R7 group*	10	1.37 (1.06, 1.78)	0.02	1.36 (1.03, 1.80)	0.03	1.52 (1.13, 2.05)	5.64 × 10^−3^	0.83 (0.41, 1.68)	0.62
	Genus. *Family* *XIIIAD3011 group*	12	0.77 (0.65, 0.90)	1.47 × 10^−3^	0.76 (0.63, 0.91)	2.74 × 10^−3^	0.87 (0.69, 1.09)	0.23	0.51 (0.22, 1.15)	0.13
OC (LGS)	Class. *Clostridia*	11	2.16 (1.19, 3.93)	0.01	2.42 (1.25, 4.67)	8.86 × 10^−3^	2.75 (1.22, 6.20)	0.01	2.70 (0.12, 60.21)	0.55
	Order. *Clostridiales*	11	2.41 (1.33, 4.37)	3.89 × 10^−3^	2.72 (1.40, 5.29)	3.13 × 10^−3^	3.21 (1.45, 7.10)	4.00 × 10^−3^	2.00 (0.10, 41.04)	0.66
	Genus. *Alloprevotella*	6	1.58 (1.13, 2.20)	7.38 × 10^−3^	1.60 (1.10, 2.33)	0.01	1.82 (1.16, 2.84)	8.94 × 10^−3^	0.83 (0.03, 21.28)	0.91
	Genus. *Collinsella*	10	0.48 (0.28, 0.84)	9.51 × 10^−3^	0.47 (0.26, 0.87)	0.02	0.61 (0.29, 1.29)	0.19	0.48 (0.06, 3.98)	0.52
	Genus. *Ruminiclostridium5*	11	2.27 (1.26, 4.07)	6.09 × 10^−3^	2.30 (1.20, 4.41)	0.01	1.76 (0.78, 3.94)	0.17	0.63 (0.06, 7.09)	0.72
OC (ED)	Family. *Streptococcaceae*	14	1.58 (1.15, 2.19)	5.19 × 10^−3^	1.59 (1.14, 2.22)	6.66 × 10^−3^	1.27 (0.81, 1.99)	0.29	0.99 (0.23, 4.25)	0.99
	Genus. *Adlercreutzia*	8	1.53 (1.15, 2.04)	3.99 × 10^−3^	1.55 (1.12, 2.14)	7.56 × 10^−3^	1.36 (0.91, 2.04)	0.14	1.82 (0.47, 7.16)	0.42
	Genus. *Ruminiclostridium6*	16	1.36 (1.07, 1.72)	0.01	1.37 (1.05, 1.77)	0.02	1.27 (0.91, 1.78)	0.16	1.00 (0.56, 1.82)	0.99
OC (IM)	Family. *Peptostreptococcaceae*	14	1.59 (1.10, 2.29)	0.01	1.61 (1.08, 2.40)	0.02	1.54 (0.93, 2.56)	0.10	1.00 (0.42, 2.39)	1.00
PC	Class. *Alphaproteobacteria*	7	0.84 (0.75, 0.93)	1.11 × 10^−3^	0.82 (0.74, 0.91)	2.32 × 10^−4^	0.84 (0.74, 0.96)	8.66 × 10^−3^	0.79 (0.51, 1.21)	0.32
	Order. *Rhodospirillales*	14	0.91 (0.85, 0.97)	6.24 × 10^−3^	0.91 (0.85, 0.98)	9.49 × 10^−3^	0.91 (0.84, 0.99)	0.03	0.73 (0.54, 0,98)	0.05
	Genus. *Adlercreutzia*	8	0.89 (0.82, 0.97)	5.18 × 10^−3^	0.88 (0.81, 0.97)	7.81 × 10^−3^	0.86 (0.77, 0.96)	8.91 × 10^−3^	0.93 (0.63, 1.37)	0.73
	Genus. *Coprobacter*	11	0.92 (0.87, 0.98)	8.28 × 10^−3^	0.92 (0.86, 0.98)	0.01	0.94 (0.87, 1.01)	0.11	1.03 (0.84, 1.26)	0.79

Abbreviations: BC, breast cancer; ER+, estrogen receptor‐positive; ER‐, estrogen receptor‐negative; EC (EH), endometrial cancer (endometrioid histology); IVW, inverse variance weighted; LC, lung cancer; LUAD, lung adenocarcinoma; LUSC, lung squamous carcinoma; OC, ovarian cancer; OC (HGS), high‐grade serous ovarian cancer; OC (LGS), low grade serous ovarian cancer; OC (ED), endometrioid ovarian cancer; OC (IM), invasive mucinous ovarian cancer; PC, prostate cancer; RAPS, robust adjusted profile score; WM, weighted median.

### 3.1 UVMR analysis

**FIGURE 2:**
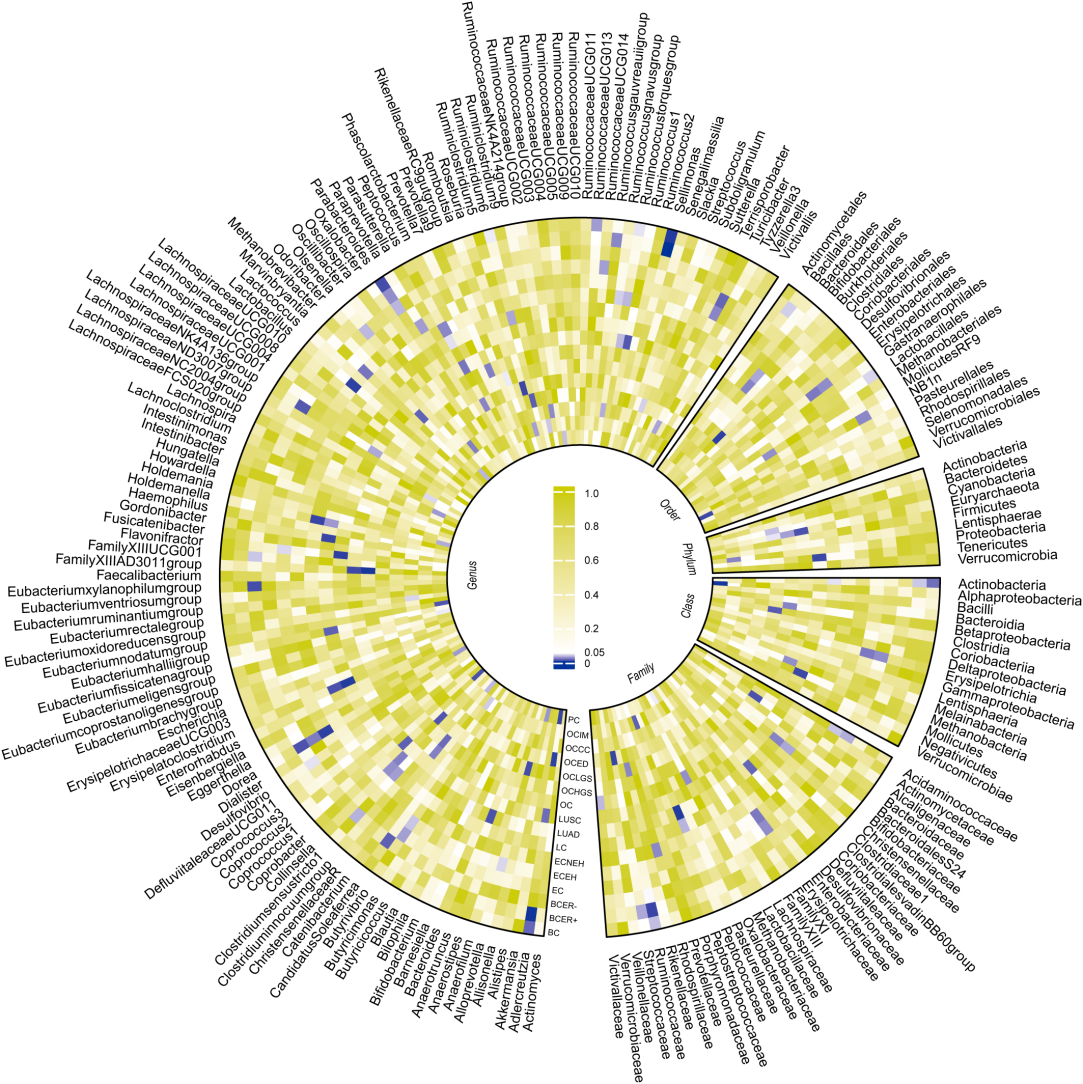
Preliminary associations between gut microbiota and cancers derived from the inverse variance weighted method. Estimates with *p* < 0.05 were shown in purple, and estimates with *p* > 0.05 were shown in white or yellow. BC, breast cancer; BCER+, breast cancer (estrogen receptor‐positive); BCER‐, breast cancer (estrogen receptor‐negative); EC, endometrial cancer; ECEH, endometrial cancer (endometrioid histology); ECNEH, endometrial cancer (non‐endometrioid histology); LC, lung cancer; LUAD, lung adenocarcinoma; LUSC, lung squamous carcinoma; OC, ovarian cancer; OCHGS, ovarian cancer (high‐grade serous); OCLGS, ovarian cancer (low grade serous); OCED, ovarian cancer (endometrioid); OCCC, ovarian cancer (clear cell); OCIM, ovarian cancer (invasive mucinous); PC, prostate cancer.

The preliminary associations between bacterial taxa at distinct taxonomic levels and the five common cancers derived from IVW were presented in Figure 2.

**FIGURE 3:**
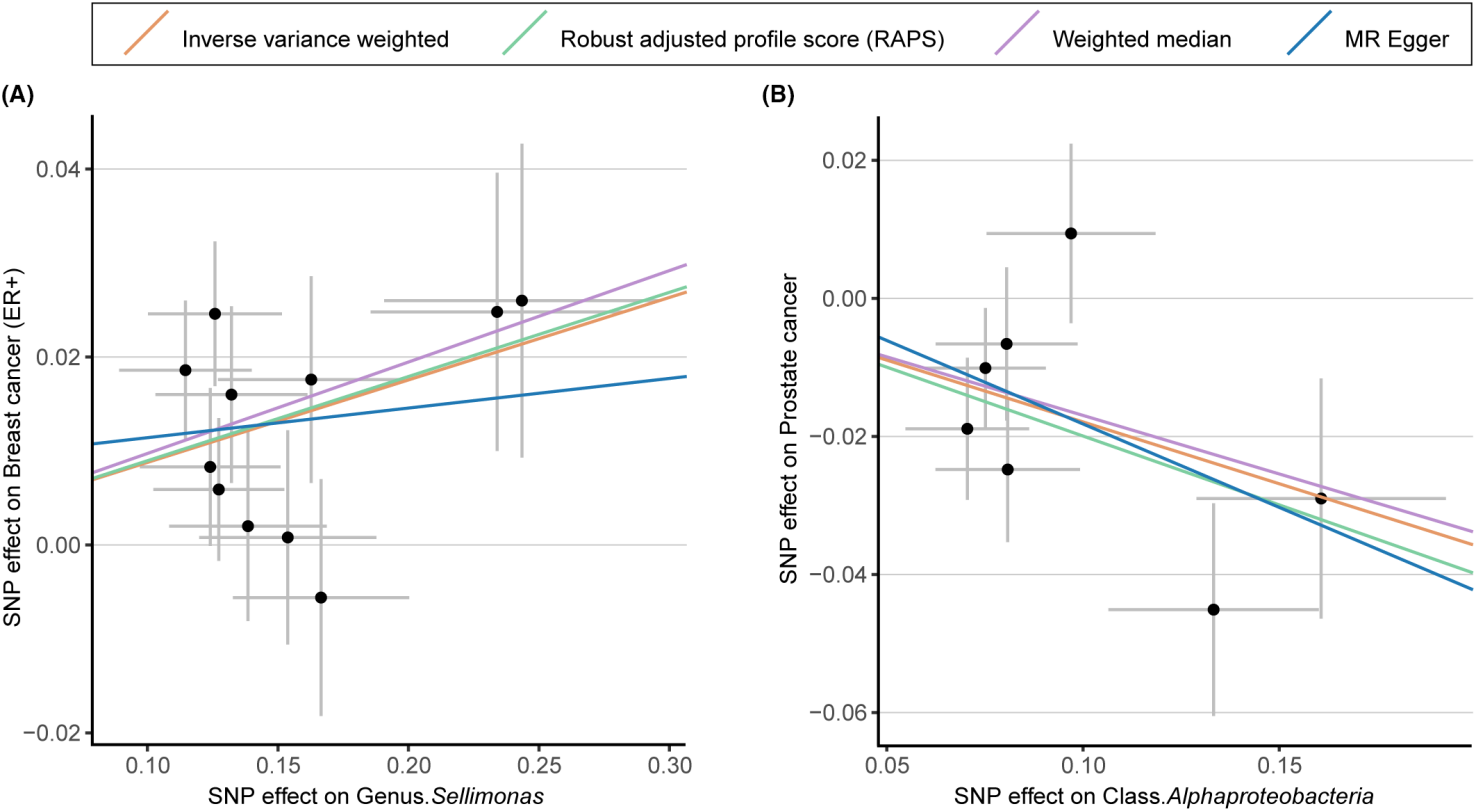
Scatterplot of two significant associations of two gut microbiota with ER+ breast cancer and prostate cancer. Scatterplot of genetic effects on genus *Sellimonas* versus the effects on ER+ breast cancer (A) and genetic effects of class *Alphaproteobacteria* versus the effects on prostate cancer (B), with corresponding standard errors denoted by horizontal and vertical lines. The slope of each line corresponds to the estimated effect from different models. ER+, estrogen receptor‐positive; SNP, single nucleotide polymorphism.

Two significant associations were identified (Table 1; Figure 3). Genetic predicted a higher abundance of genus *Sellimonas* was significantly associated with an increased risk of ER+ BC (IVW OR = 1.09, 95% CI 1.05–1.14, *p* = 2.01 × 10^−5^). We also observed that a genetically predicted higher abundance of class *Alphaproteobacteria* was causally associated with a decreased risk of PC (IVW OR = 0.84, 95% CI 0.75–0.93, *p* = 1.11 × 10^−3^). The consistent direction and magnitude of the estimates from other MR models, including RAPS, WM, and MR‐Egger regression, further supported the causal inferences (Table 1). Cochran Q test indicated no heterogeneity was detected (Table S2). MR‐Egger intercept analysis suggested that there was no potential horizontal pleiotropy (Table S2). LOO analysis further supported that the causalities were not driven by any single SNP (Figure S1).

In addition, there were also suggestive causal effects of phylum *Tenericutes*, classes (*Actinobacteria*, *Mollicutes*, *Clostridia*), orders (*Clostridiales*, *Rhodospirillales*), families (*Peptococcaceae*, *Alcaligenaceae*, *Streptococcaceae*, *Peptostreptococcaceae*), and genera (*Adlercreutzia*, *Alloprevotella*, *Christensenellaceae R7 group*, *Collinsella*, *Coprobacter*, *Dorea*, *Eubacterium Hallii group*, *Eubacterium xylanophilum group*, *Family XIIIAD3011 group*, *Flavonifractor*, *Gordonibacter*, *Holdemanella*, *Lachnospiraceae NK4A136 group*, *Lactobacillus Erysipelatoclostridium*, *Parabacteroides*, *Ruminiclostridium 5*, *Ruminiclostridium 6*, *Ruminococcus 1*, *Sellimonas*) on at least one of the cancers (Table 1). No heterogeneity or pleiotropy was detected in the sensitivity analysis (Table S2).

### 3.2 MVMR analysis

**FIGURE 4:**
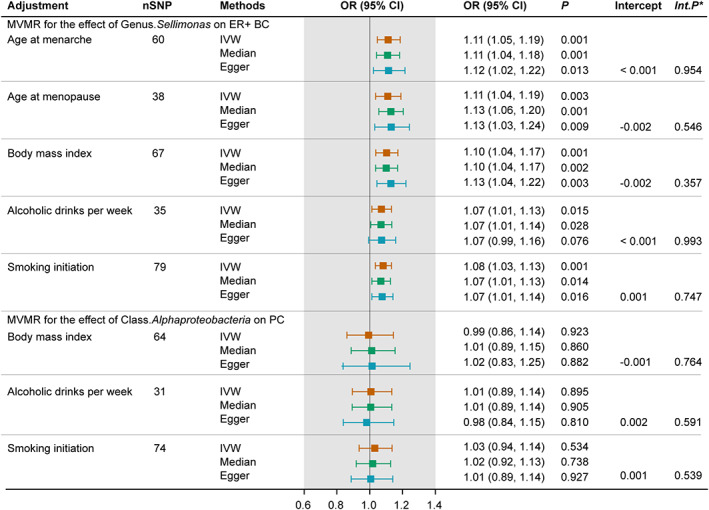
Forest plot for the MVMR accounting for common risk factors of breast and prostate cancer. *Int.*p* refers to the *p* values derived from Egger intercepts. CI, confidence interval; ER+ BC, estrogen receptor‐positive breast cancer; IVW, inverse variance weighted; OR, odds ratio; PC, prostate cancer; SNP, single nucleotide polymorphism.

To determine whether genus *Sellimonas* or class *Alphaproteobacteria* exerted an impact on cancer risk directly or through common cancer risk factors, we further conducted an MVMR analysis. The effect of genetically predicted genus *Sellimonas* on ER+ BC remained after accounting for age at menarche, age at menopause, BMI, alcoholic drinks per week, and regular smoking (Figure 4). The causal inference was further supported by consistent direction and magnitude from distinct MR models. Besides, the intercept term derived from MR‐Egger did not detect potential horizontal pleiotropy.

However, the association between genetic predisposition toward class *Alphaproteobacteria* and PC was attenuated with adjustment of BMI, alcoholic drinks per week, and regular smoking. The Egger intercept test indicated low risks of bias owing to horizontal pleiotropy.

## 4 DISCUSSION

Using MR design, we investigated the potential causal association between genetically proxied gut microbiota and five common cancers, including BC, EC, LC, OC, and PC. We found that genetically predicted a higher abundance of genus *Sellimonas* directly exerted a detrimental effect on the risk of ER+ BC after accounting for age at menarche, age at menopause, BMI, alcoholic drinks per week, and regular smoking; and that the protective role of a higher abundance of class *Alphaproteobacteria* on PC development might be driven BMI, drinking and smoking behaviors. Besides, nominal significant results also implicated the heterogeneous impacts of gut microbiota on different cancers. To our knowledge, this is the first MR study to comprehensively investigate the role of gut microbiota in the issue of cancer risk in a causal way.

The associations of gut microbiota with cancers have been noted. The vast majority of literature focused on the involvement of gut microbiota in gastrointestinal cancers as they share the same ecosystem, thereby prone to speculating potential relationships existing between them. However, accumulation of evidence endorsed a potential link between gut microbiota and cancers of other systems. Zheng et al. have reported that patients with lung cancer presented a significant shift in microbiota composition compared with controls.^50^ Zhu et al. also observed alteration of the gut microbial community in breast cancer patients.^51^ Undoubtedly, the role of gut microbiota in cancer development is catching more attention. However, there is a paucity of convincing evidence on a cancer‐causing gut microbial composition. Even though a few mechanisms have been proposed as the potential pathways from the gut microbiota to oncogenesis in animal models, the exact causality between gut microbiota and human cancer risk could not be fully determined owing to the intricate interaction between gut flora and the human host. Previous conventional observational studies have also struggled to decipher the secrets of gut microbiota in modulating human health, but the inherent methodologic defects make the exact causal effect remains elucidating. Besides, intestinal bacterial communities can be disturbed by various factors, like diet, drugs, diseases, and other environmental factors,^52^ which would make the observed association of gut microbiota with cancers not convincing. Taken together, the role of gut microbiota in cancer development remains to be explored by researchers. Inspired by the application of large‐scale GWAS, which enabled us to utilize summary‐level statistics for causal inference, this work concentrated on the causalities between gut microbiota and several common cancers and hoped to find some evidence of the existence of the axis linking the gut with other systems.

Using MR design, we found that genetically proxied higher abundance of genus *Sellimonas* predicted a higher risk of ER+ BC. Besides, further MVMR analysis indicated a robust causality between genus *Sellimonas* and ER+ BC after accounting for age at menarche, age at menopause, BMI, smoking, and drinking behaviors. This strongly implicated that such a detrimental effect was, at least partially, independent of the common risk factors of BC. Previously, literature about *Sellimonas* was extremely limited. It has been reported *Sellimonas* was overrepresented in fecal specimens from patients with more aggressive tumors, suggesting a potential carcinogenic role of *Sellimonas* in human hosts. However, the underlying mechanism warrants future investigation. Further pathways from *Sellimonas* to ER+ BC could be expected in future studies.

For PC, we observed a protective role of class *Alphaproteobacteria* in PC, which was concordant with previous studies. MVMR analysis further revealed that the protective effect of *Alphaproteobacteria* on PC might be driven by BMI, smoking, and drinking behaviors. A previous study has reported that extracts from the member of *Alphaproteobacteria* could attenuate benign prostate hyperplasia that would potentially increase the risk of PC.^53^ Another member of *Alphaproteobacteria* was reported to produce glionitrin B with an anti‐invasion effect of PC when cocultured with another fungus.^54^ In terms of mechanisms, Sookoian et al. have reported that compared with morbidly obese patients, non‐morbidly obese controls represented a higher abundance of *Alphaproteobacteria*, which might potentially mediate the occurrence of PC.^55^ Notwithstanding, the links between *Alphaproteobacteria* and PC still warrant further investigation.

Our study should be evaluated in light of several implications. First, in the absence of RCT, our work expanded the current literature on the issue of the causal association between gut microbiota and cancers and provided robust evidence. Second, we would rather obtain indicators of disease risk from MR results than extrapolate MR results to an expected effect from intervention in clinical sets, which has been proposed by methodologists.^37^, ^56^ From this perspective, the main finding of our study implicated that stool examination might be a feasible strategy to identify populations at higher risk of BC and PC and to further advocate for more frequent cancer screening or undertaking more thorough examinations. Third, except for the significant estimates aforementioned, the current study also identified nominal significant associations between a range of gut microbiota and cancers. While the observed associations did not reach Bonferroni correction significance, the potential impact of these gut microbiota should not be ignored. Instead, these results might point to a potential cancer‐causing bacterial composition that would help in evaluating cancer risk and provide candidate bacteria that might have an implication for investigators to focus on certain specific gut microbiota in future functional studies.

Some limitations should be noted in this study. The first limitation that should be pointed out is that the major participants of our study were constrained to European ancestry. While this would limit bias owing to population heterogeneity, whether the MR results are general in other populations warrants future investigations. Second, we relaxed the *p* threshold between instruments and exposures to obtain a larger number of SNPs, which might increase the risk of violating the first assumption of MR design. However, the F statistic for each SNP was over 10, indicating that no weak SNPs were included for MR estimation. Besides, the significant results were identified based on rigorous Bonferroni correction to lower the risk of false‐positive results. Third, we failed to fully mitigate pleiotropy as specific biologic functions of the employed SNPs remain unknown to date. However, it is reassuring distinct MR models presented concordant estimates and sensitivity analyses based on various assumptions failed to detect any horizontal pleiotropy.

In conclusion, this MR study sheds light on a potential causal role of gut microbiota in cancer development. This would have an implication to clinicians that early stool examination might be a feasible practice for cancer screening to recognize populations at a higher risk of cancer, and in addition, conditioning of gut microbiota might be a potential treatment for cancer prevention. Future work is warranted to decipher the underlying mechanism.

## AUTHOR CONTRIBUTIONS

**Zixin Wei:** Conceptualization (equal); formal analysis (equal); methodology (equal); software (equal); validation (equal); writing – original draft (equal); writing – review and editing (equal). **Biying Yang:** Conceptualization (equal); methodology (equal); writing – original draft (equal); writing – review and editing (equal). **Tiantian Tang:** Investigation (equal); methodology (equal); software (equal); supervision (equal); validation (equal); writing – review and editing (equal). **Zijing Xiao:** Validation (equal); visualization (equal); writing – review and editing (equal). **Fengzhan Ye:** Visualization (equal); writing – review and editing (equal). **Xiaoyu Li:** Validation (equal); writing – review and editing (equal). **Shangbin Wu:** Supervision (equal); validation (equal); writing – review and editing (equal). **Jin‐gang Huang:** Supervision (equal); writing – review and editing (equal). **Shanping Jiang:** Conceptualization (equal); data curation (equal); project administration (lead); writing – review and editing (equal).

## CONFLICT OF INTEREST STATEMENT

The authors declare no conflicts of interest.

## ETHICS APPROVAL

In the present study, we only utilized publicly available summary data, and the Ethics approval and consent to participants could be obtained in the original GWAS.

## Supporting information


Figure S1.

Table S1–S2.

## Data Availability

The main text and supplementary materials have included all the data generated in the present study. The sources of the GWAS statistics used in this study could be found in the original GWAS.
