# Supplementary material for: Gut microbiota and risk of five common cancers: A univariable and multivariable Mendelian randomization study
Source: Cancer Med. 2023 Mar 7;12(9):10393–405. doi: 10.1002/cam4.5772 (PMC10225193; doi:10.1002/cam4.5772)

**Figure S1.** Forest plots for the Mendelian randomization (MR) leave one out analysis of the significant and nominal significant results.

Within each panel, the black points represent the causal estimate of association between a specific exposure and target mental disorder after discarding each SNP in turn. Red points represent the overall causal estimate using the random-effects inverse variance weighted. Horizontal lines denote 95% confidence intervals.

BC, breast cancer; BCER+, breast cancer (estrogen receptor-positive); BCER-, breast cancer (estrogen receptor-negative); ECEH, endometrial cancer (endometrioid histology); LC, lung cancer; LUAD, lung adenocarcinoma; LUSC, lung squamous carcinoma; OC, ovarian cancer; OCHGS, ovarian cancer (high grade serous); OCLGS, ovarian cancer (low grade serous); OCED, ovarian cancer (endometrioid); OCIM, ovarian cancer (invasive mucinous); PC (prostate cancer).


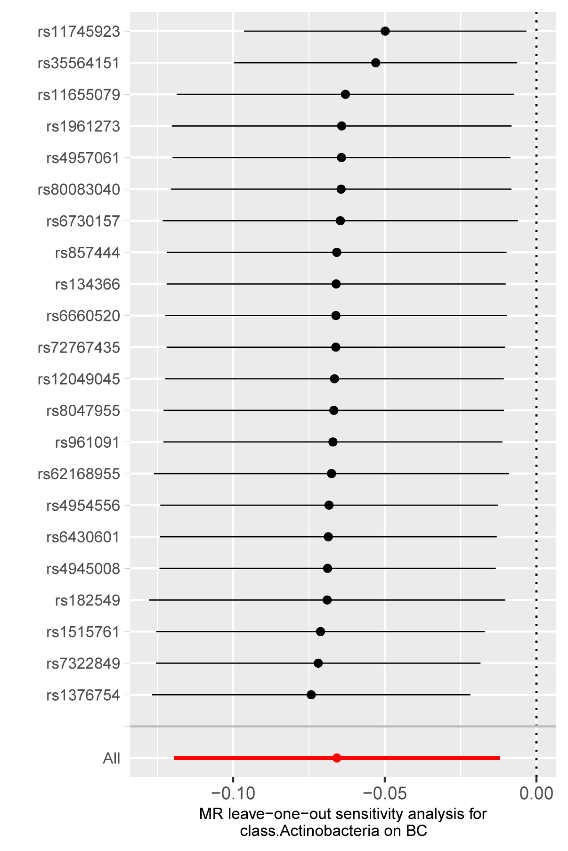

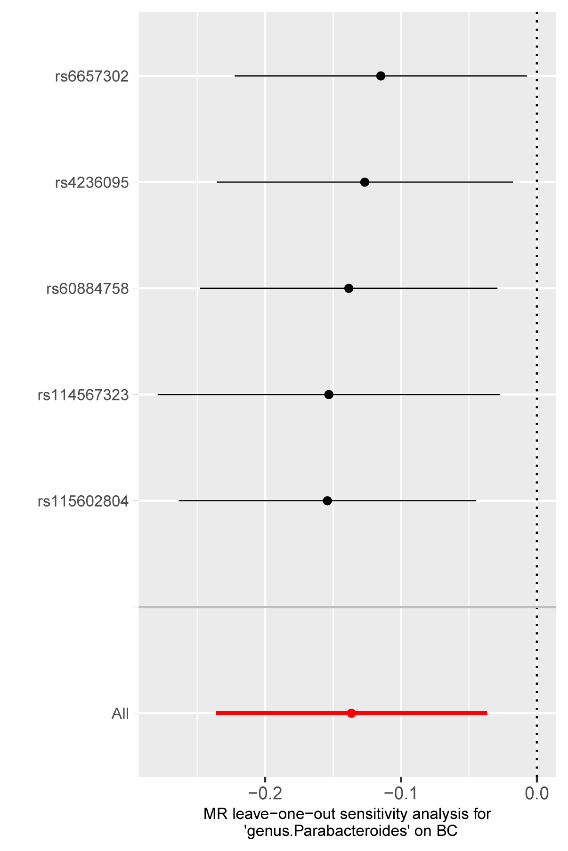


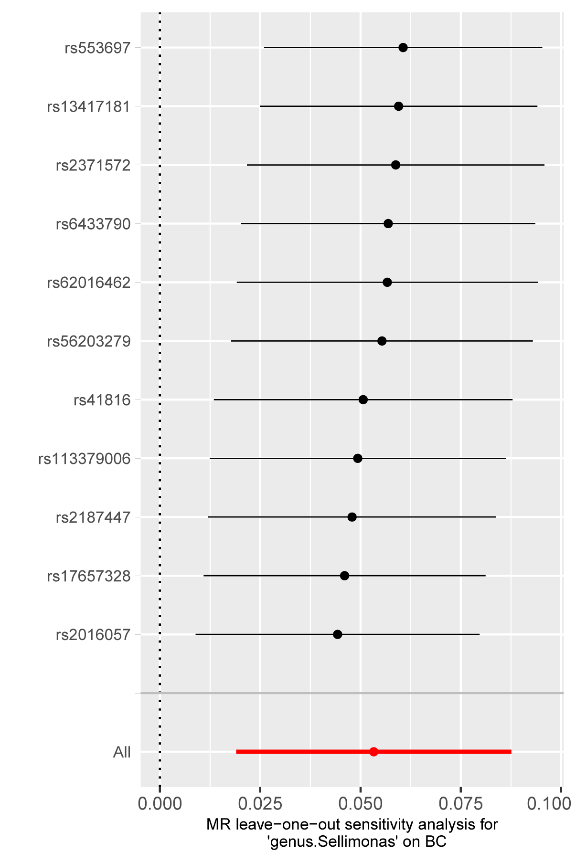

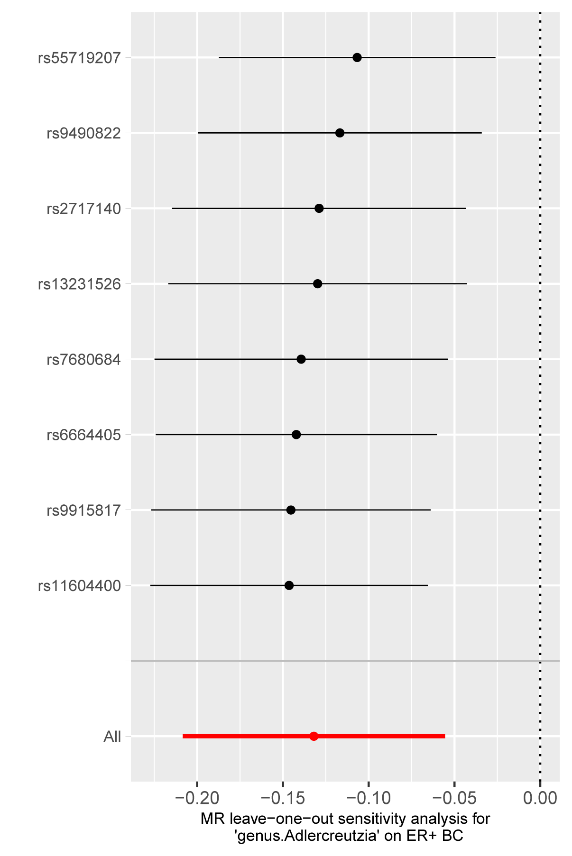

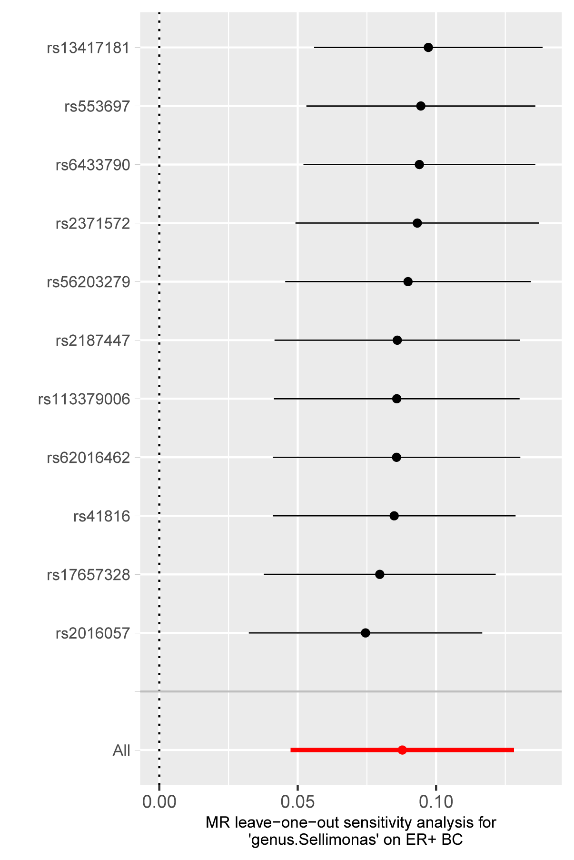

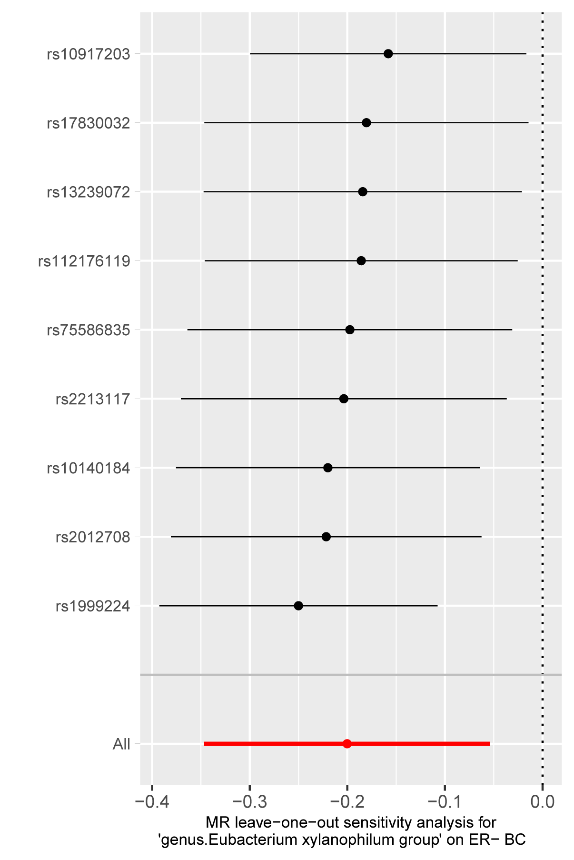


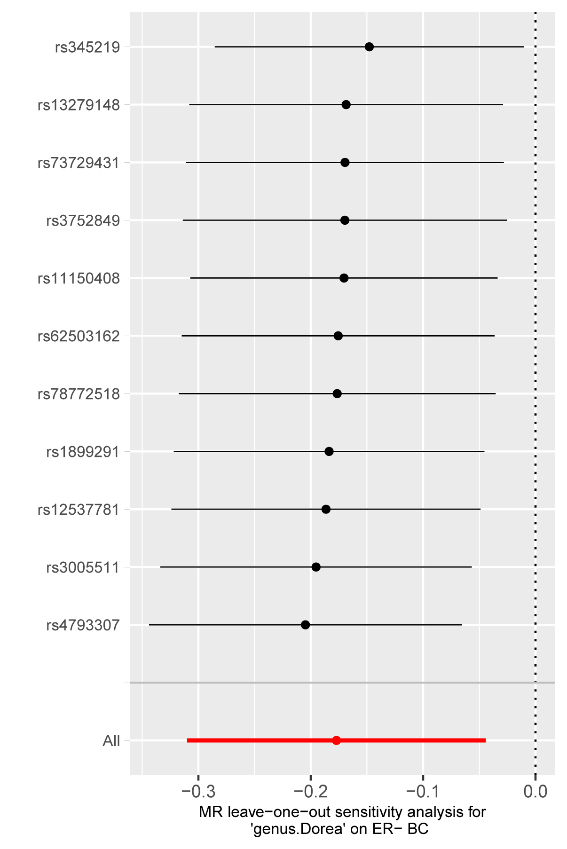

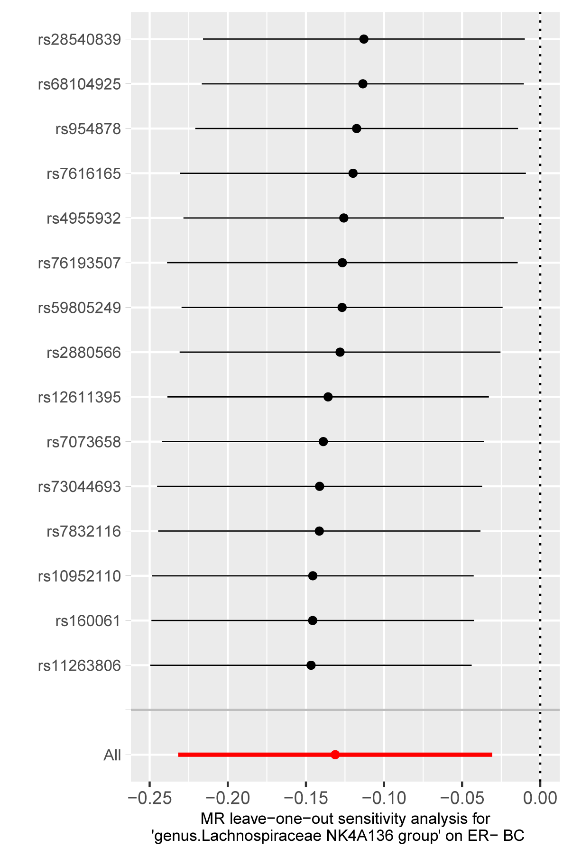

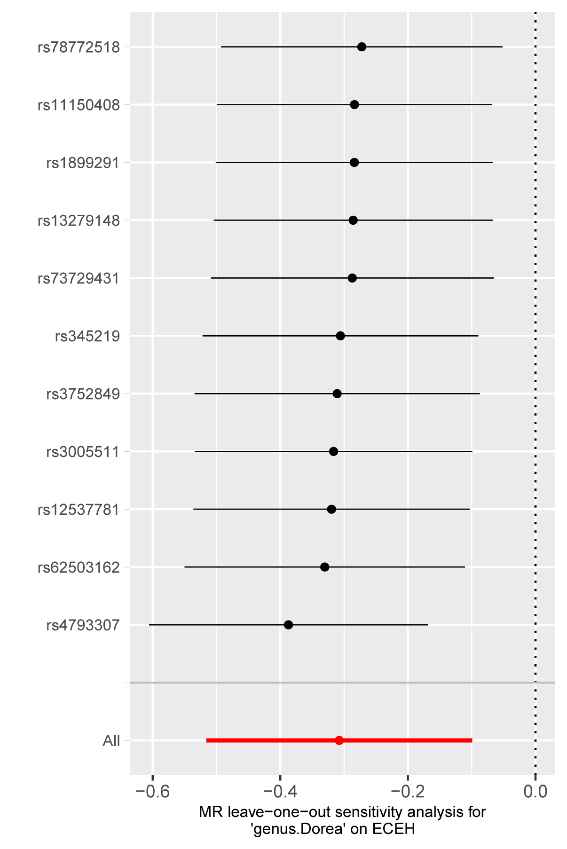

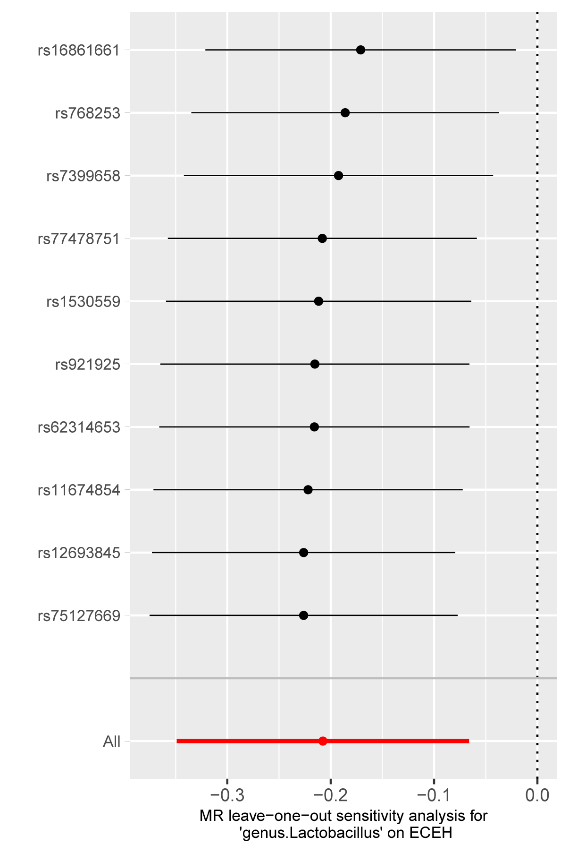


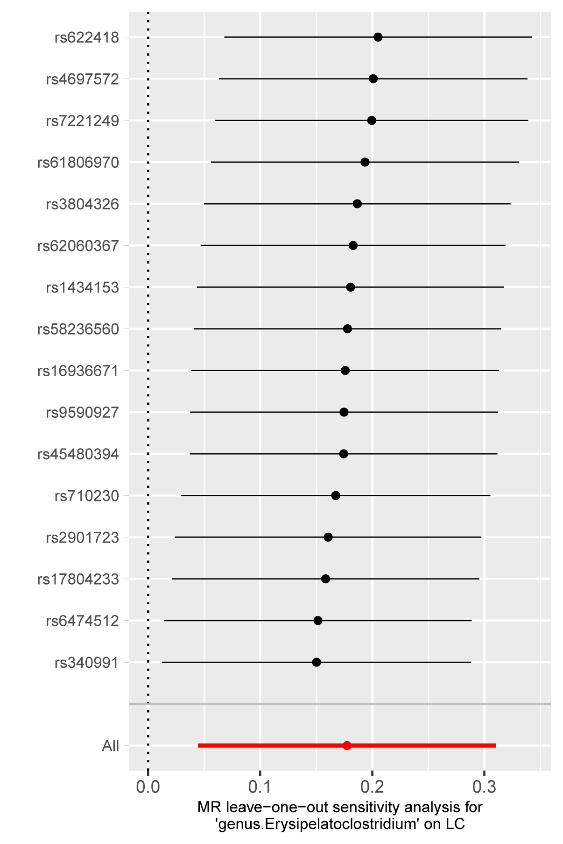

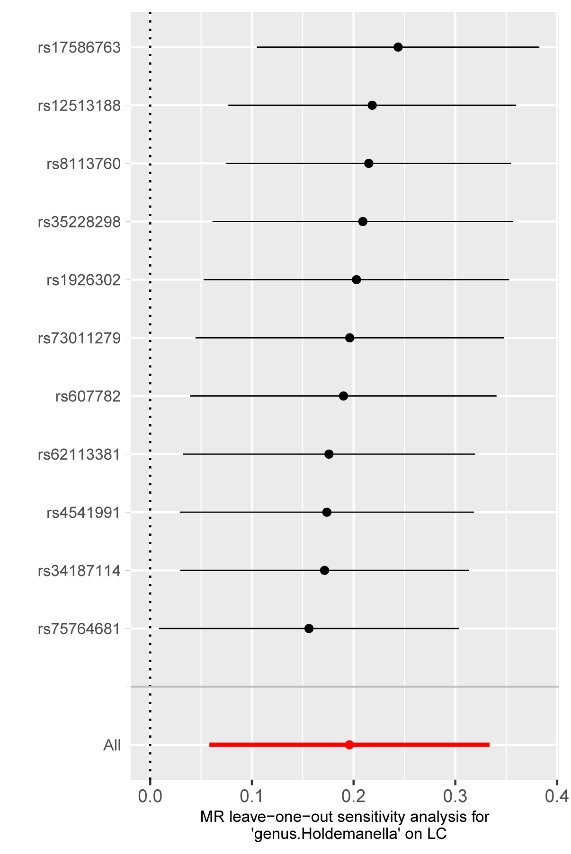

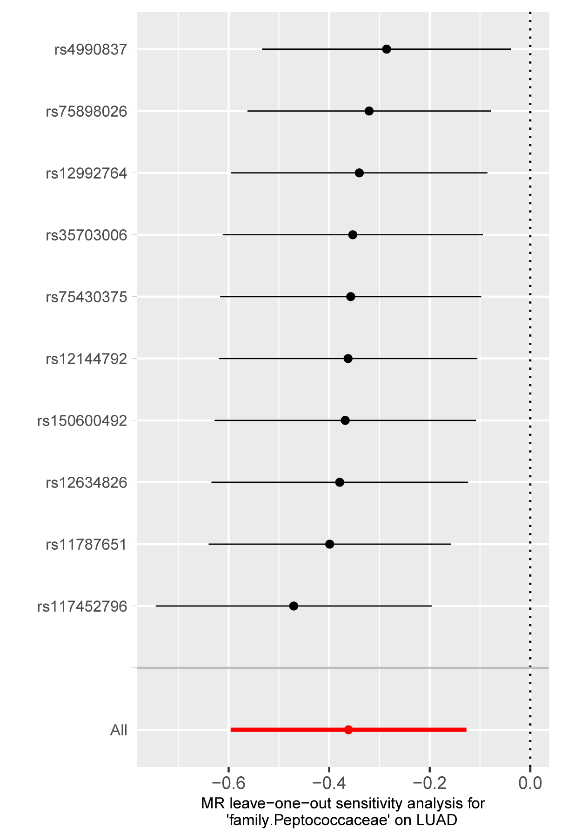

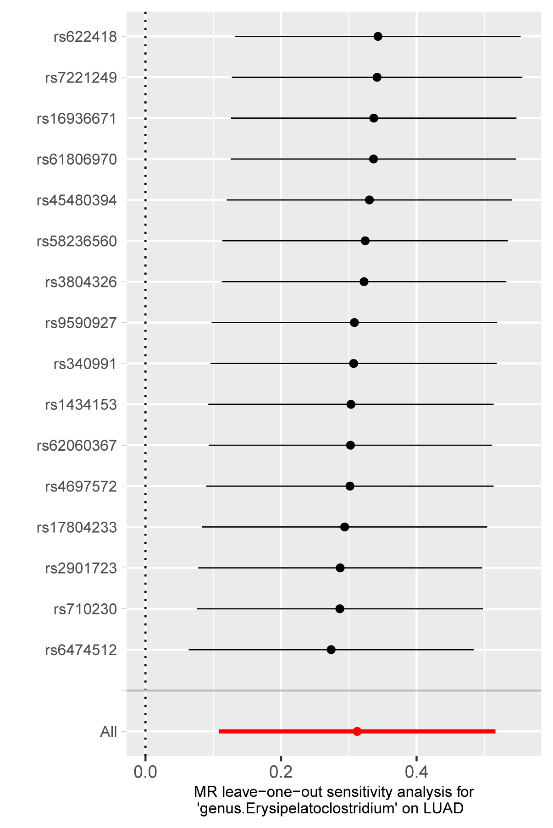


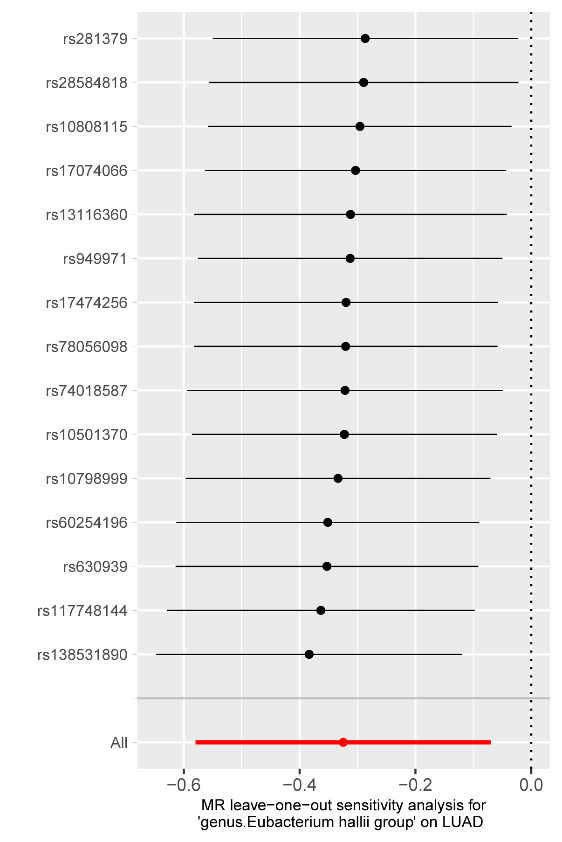

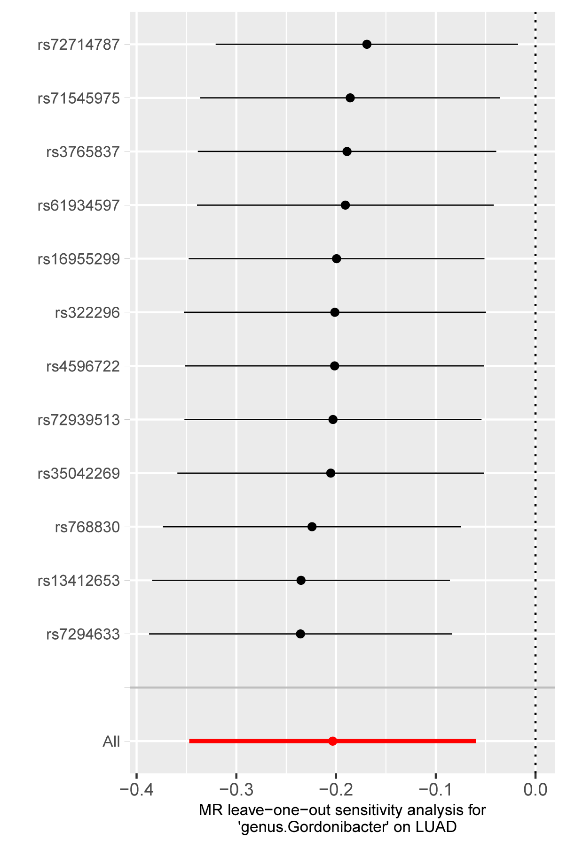

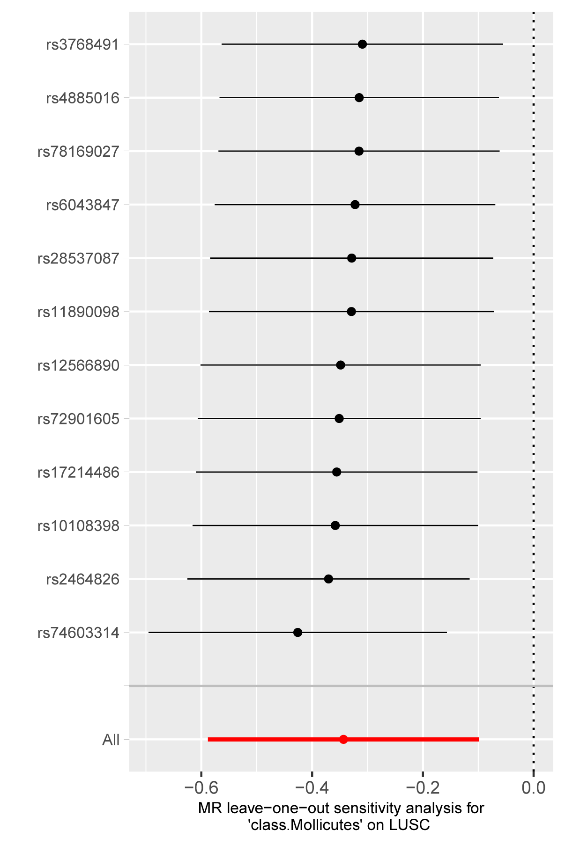

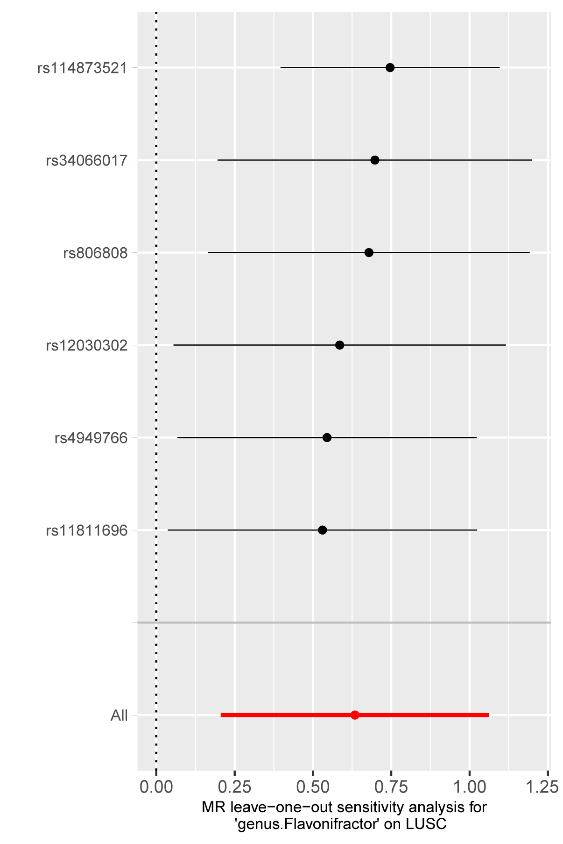


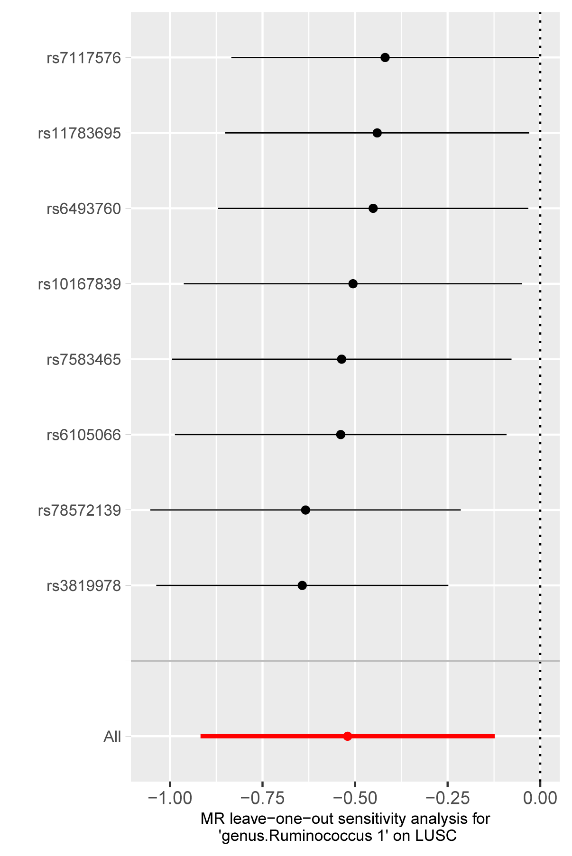

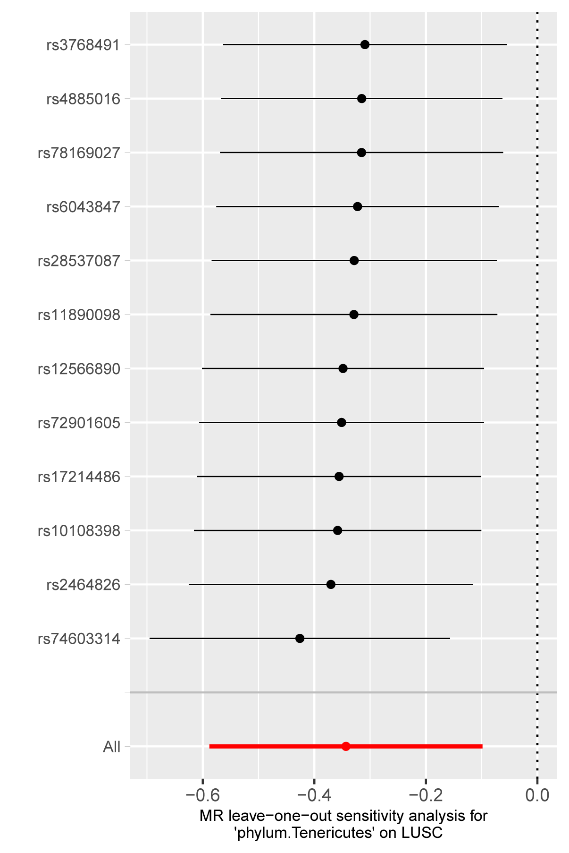

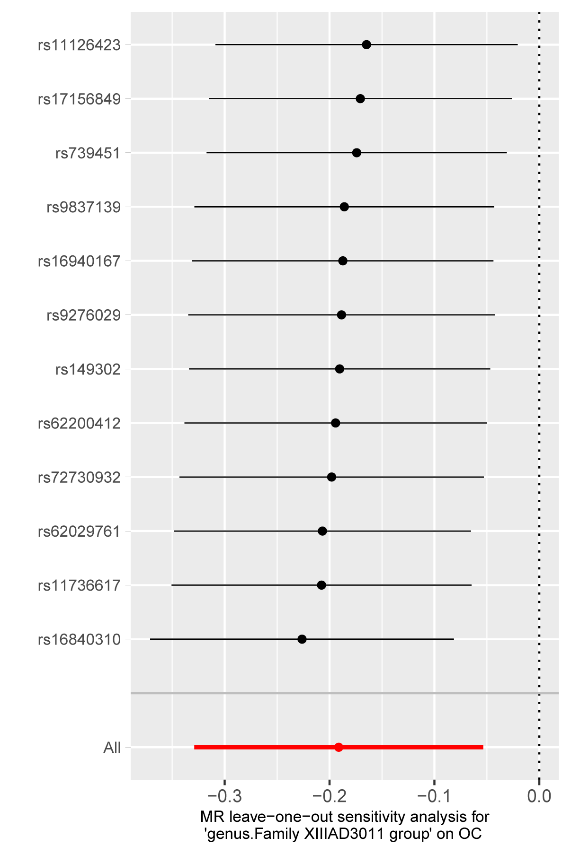

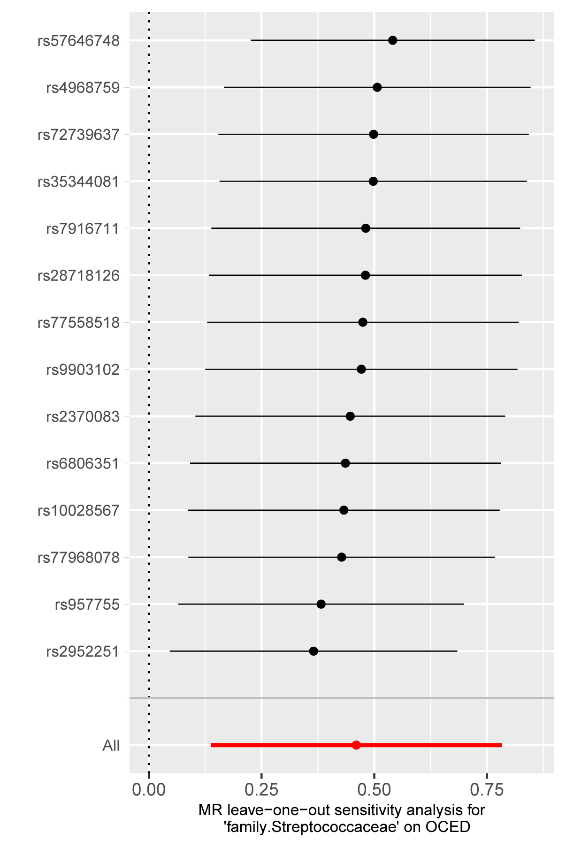


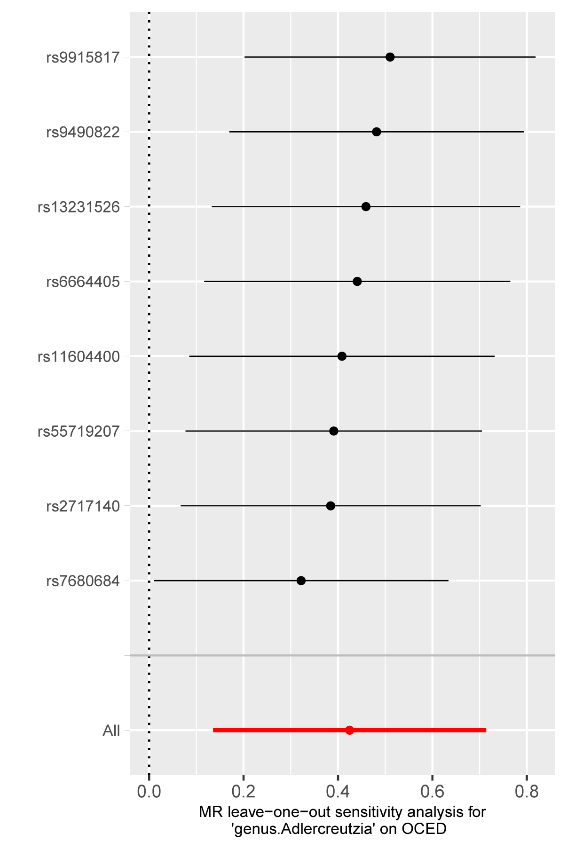

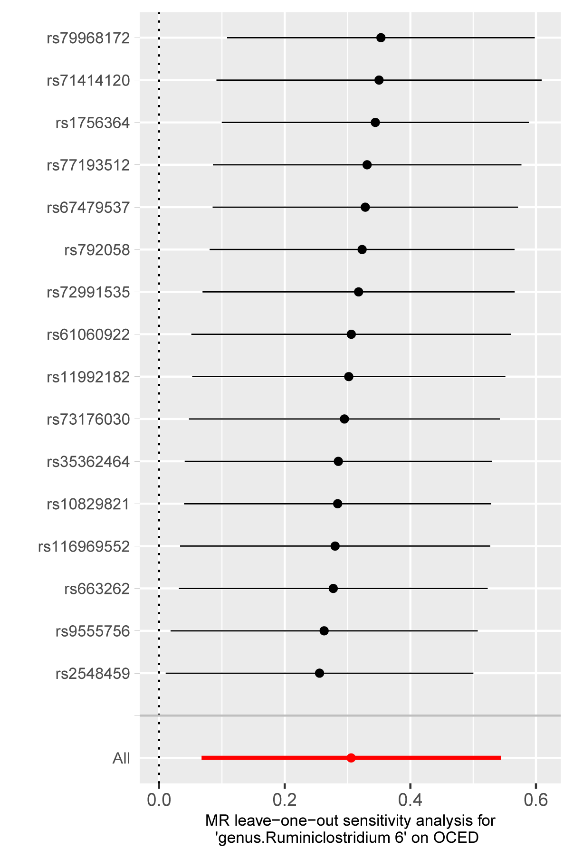

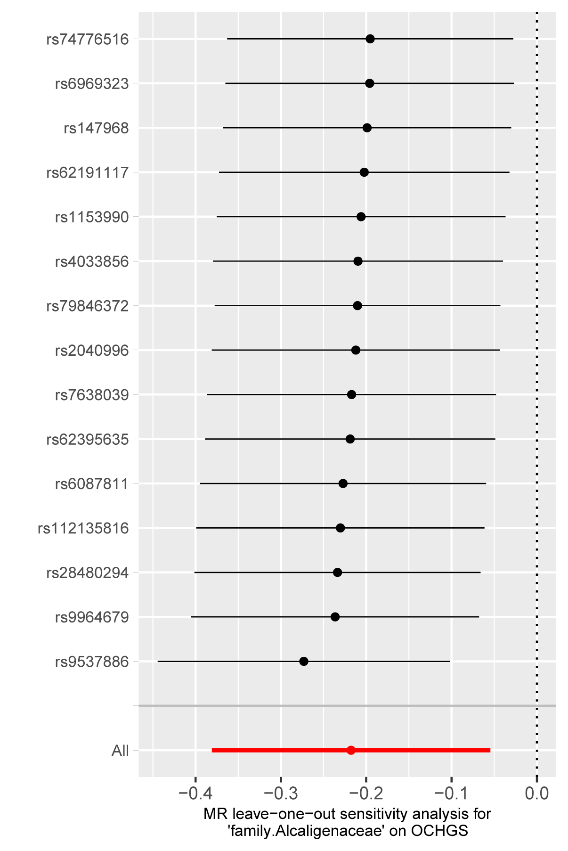

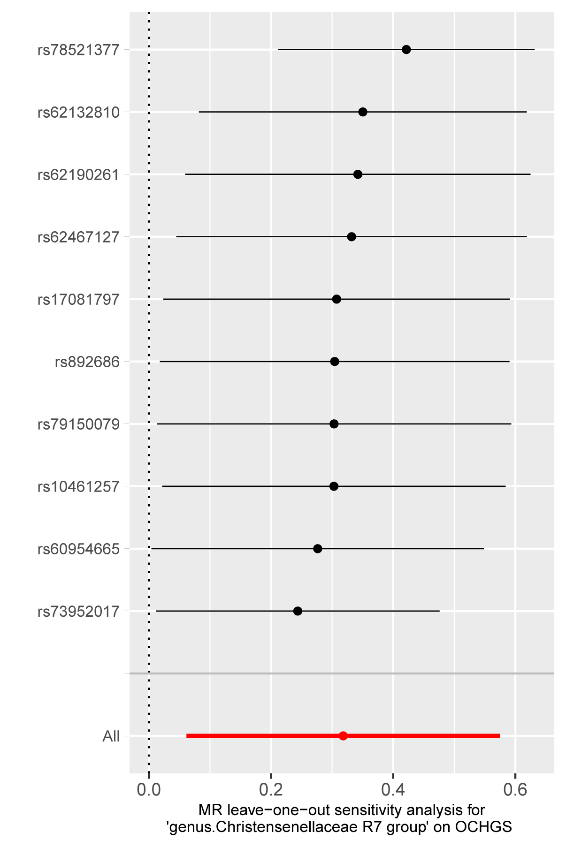


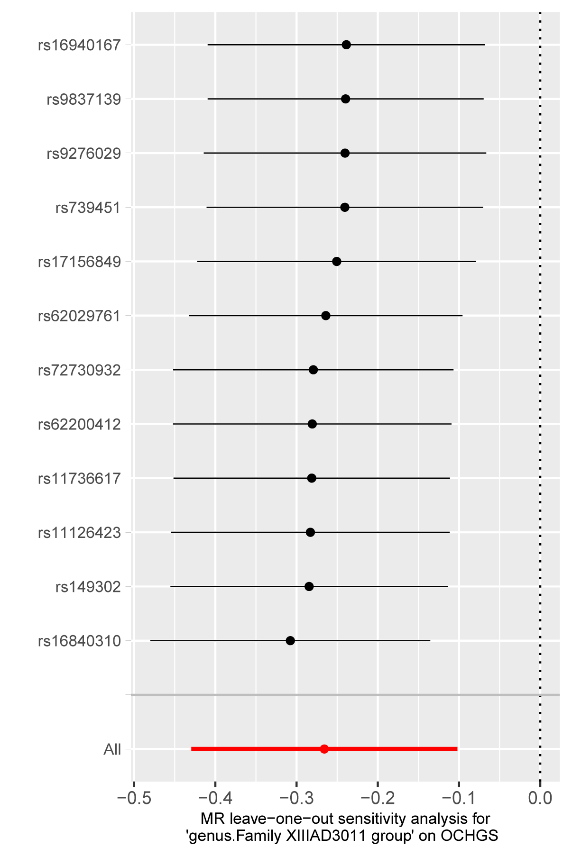

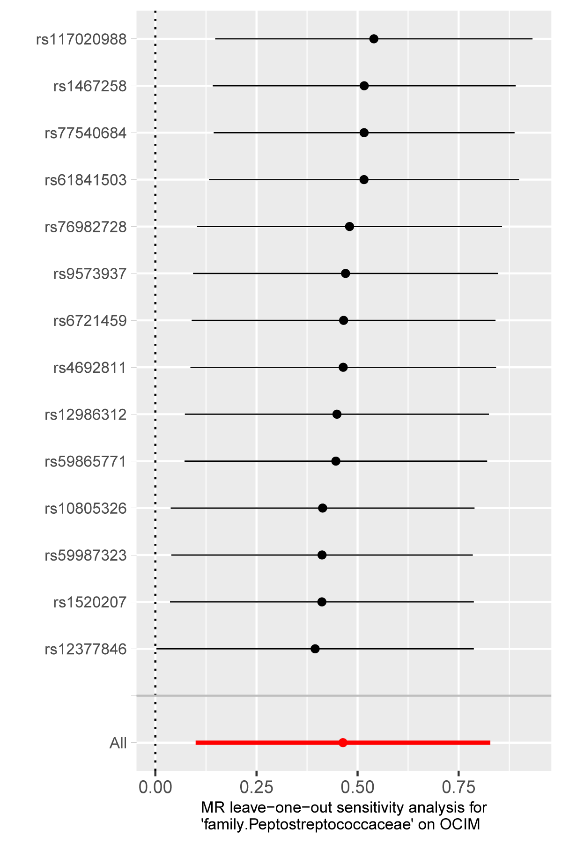

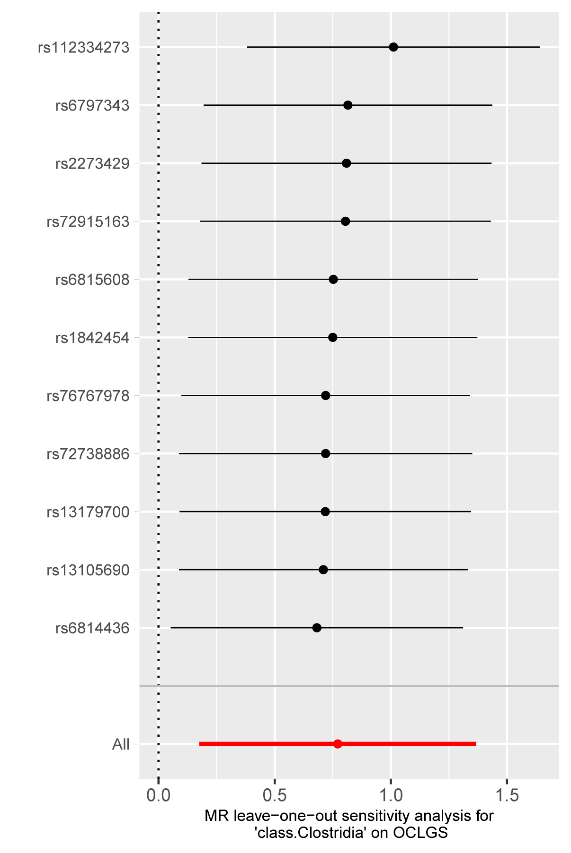

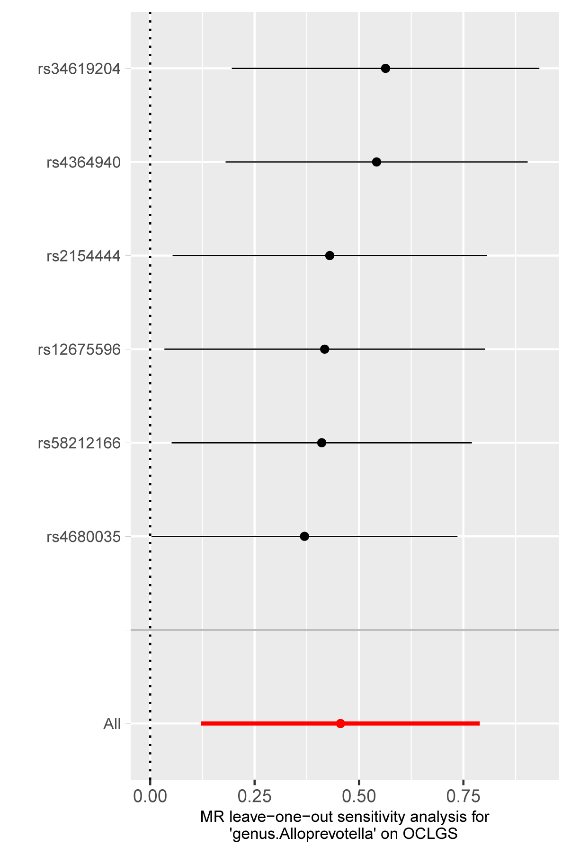


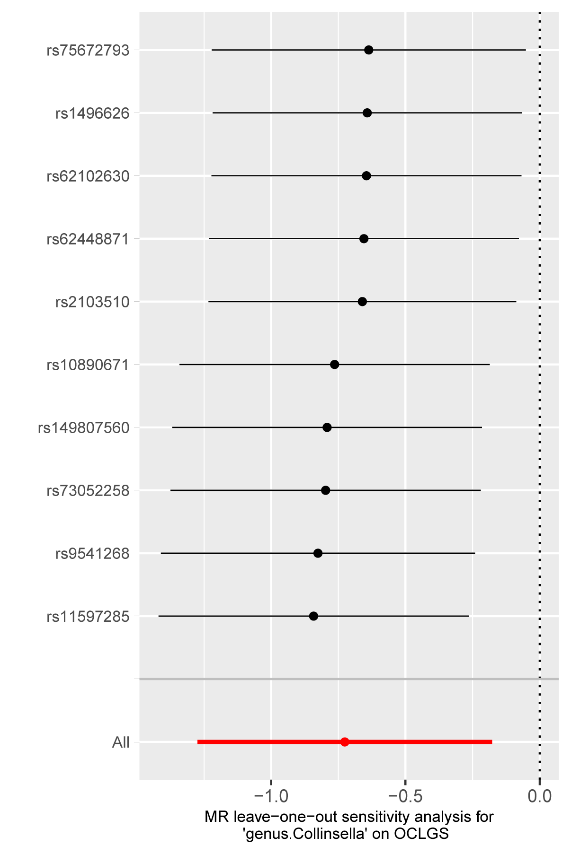

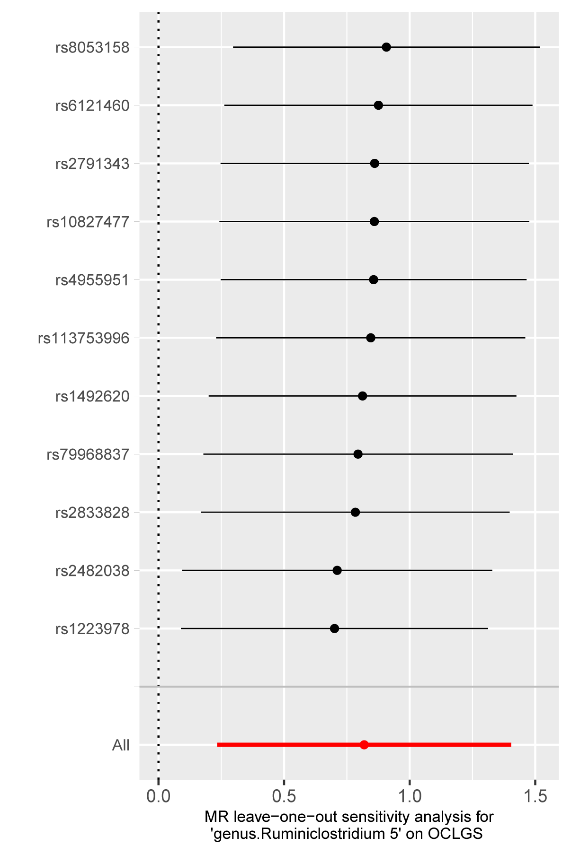

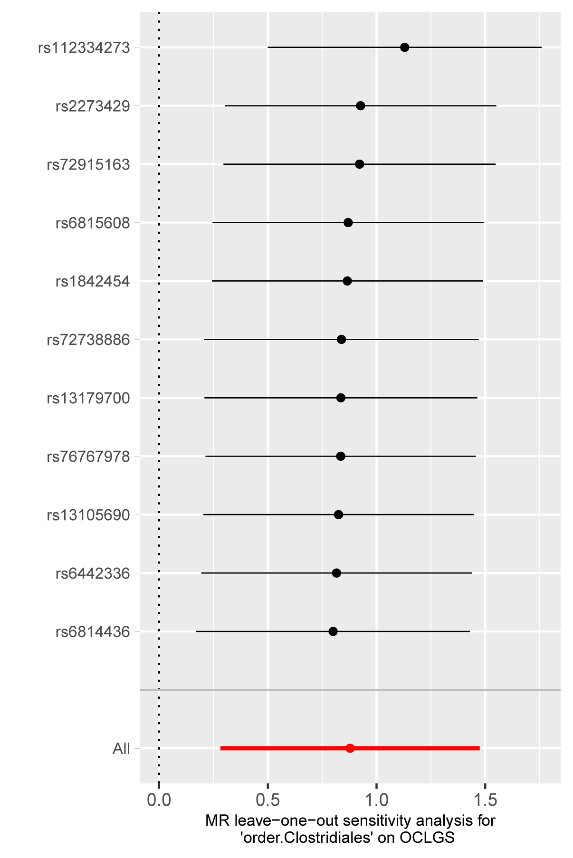

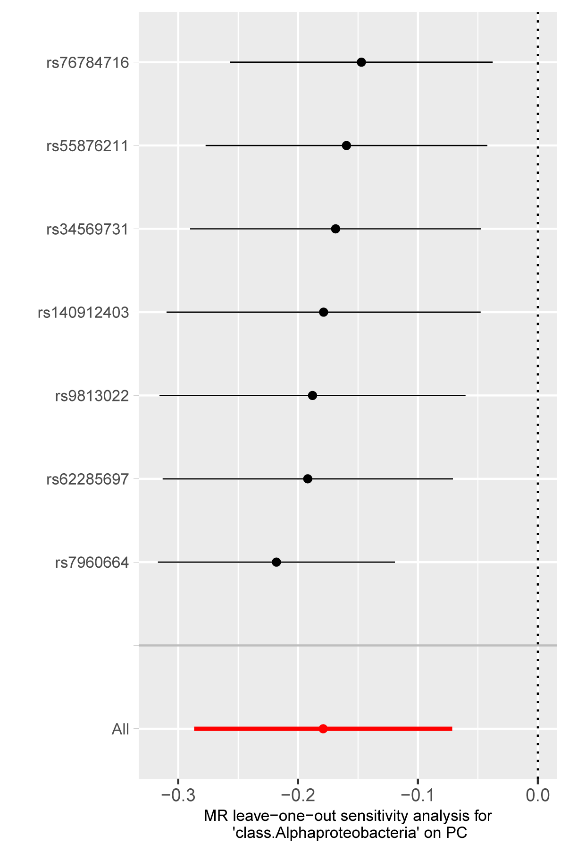


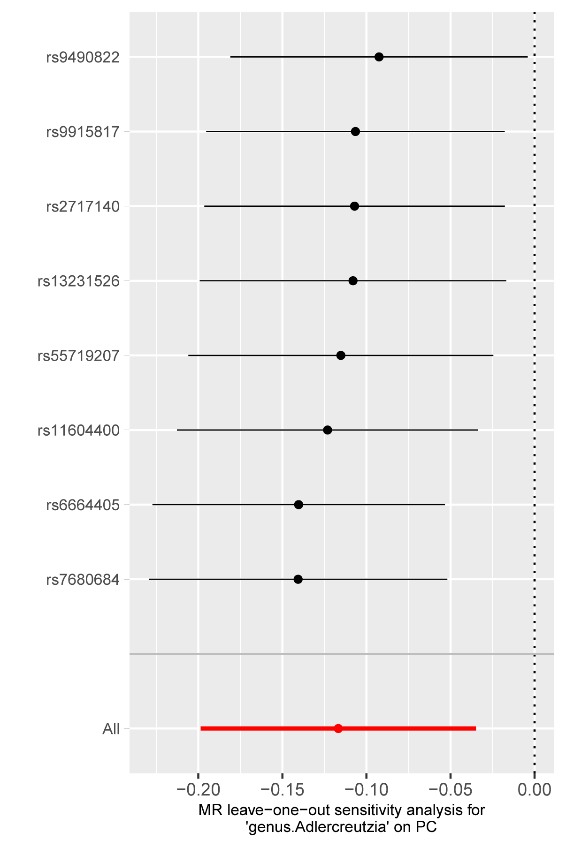

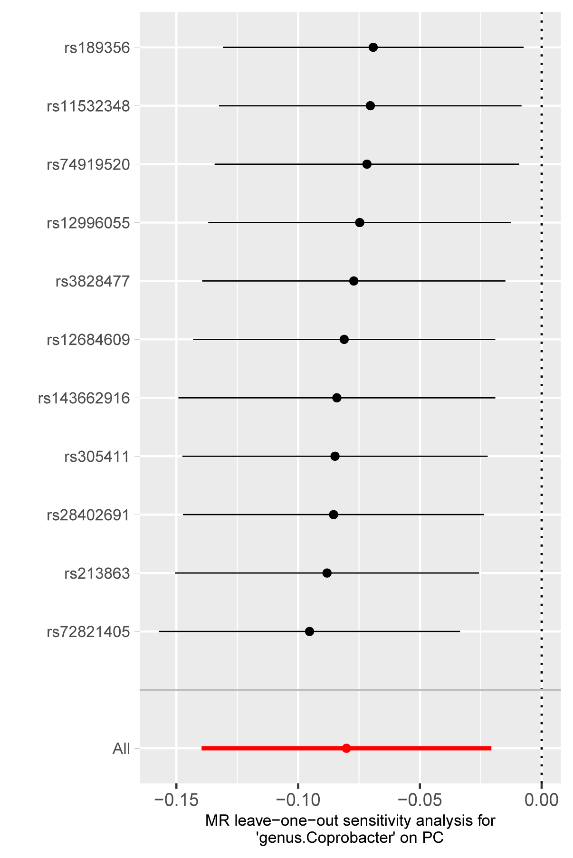

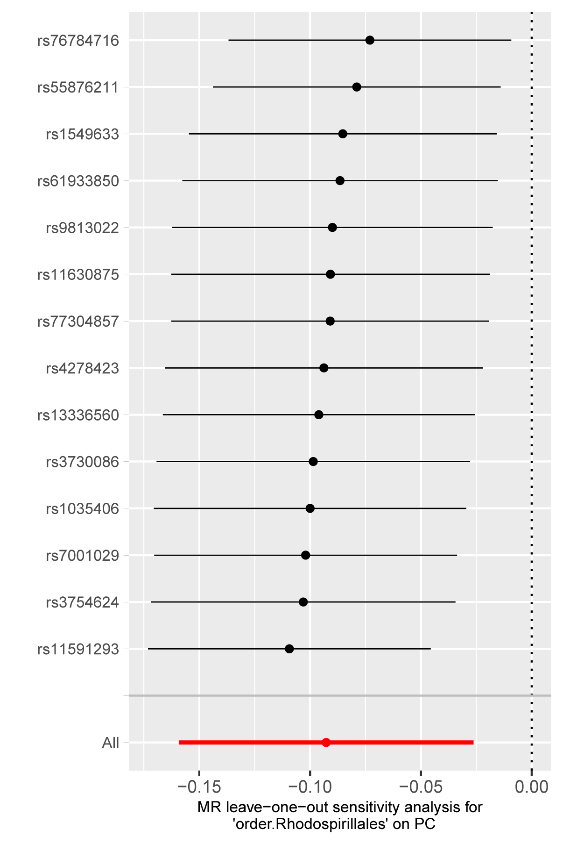

Supplement: Supplementary file 1 — Figure S1. [file CAM4-12-10393-s001.docx]
